# Carbazole‐Based Thin Microporous Polymer Films for Photocatalytic Hydrogen Evolution

**DOI:** 10.1002/adma.202506689

**Published:** 2025-06-23

**Authors:** Veit Dippold, Hüseyin Küçükkeçeci, Eugenia Bosler, Johannes Schmidt, Samrat Ghosh, Gregor Michl, Islam E. Khalil, Lisa Gerland, Adam Lange, Dirk Oberschmidt, Arne Thomas

**Affiliations:** ^1^ Department of Chemistry/Functional Materials Technische Universität Berlin Hardenbergstr. 40 10623 Berlin Germany; ^2^ Department of Micro and Precision Devices Technische Universität Berlin Pascalstraße 8‐9 10587 Berlin Germany; ^3^ Research Unit Molecular Biophysics Leibniz‐Forschungsinstitut für Molekulare Pharmakologie Robert‐Roessle‐Str. 10 13125 Berlin Germany

**Keywords:** carbazoles, films, hydrogen evolution, laser ablation, photocatalysis, polymers

## Abstract

Photocatalytic hydrogen evolution is a direct pathway to store solar energy in chemicals. Conjugated microporous polymers (CMPs) are porous organic photocatalysts, that are typically applied in powder form in heterogenous catalytic reactions. However, the use of powder photocatalysts in dispersion poses some major challenges when it comes to practical applications in larger scales. In this manuscript, the photocatalytic performance of a carbazole‐based porous organic polymer (C‐POP) film produced by electro polymerizing 1,2,3,5‐Tetrakis(carbazol‐9‐yl)‐4,6‐dicyanobenzene (4CzIPN) is investigated, well‐known for its intriguing photocatalytic properties. The thickness of the intrinsic microporous film is tuneable by the amount of cyclic voltammetry cycles but it is shown that the hydrogen production is not dependent on film thickness. It can therefore be concluded that catalysis is mainly occuring on the outer surface of the films, questioning whether high surface areas are always required for efficient photocatalysis. A microstructured film offers the advantage that, with a reduced amount of polymer material, a constant or even increased external surface area of the film can be achieved. The approach presented here is therefore advantageous for achieving high hydrogen production per unit area with minimal amounts of polymer, as very thin layers are already sufficient for high activity.

## Graphical abstract

Photocatalytic hydrogen evolution with electropolymerized microporous films enable a direct and scalable process for the production of industrial relevant amounts of hydrogen gas. For developing cost‐ and material‐effective devices it is beneficial to increase the accessible outer surface area via microstructuring and stacking of polymeric films instead of using thick polymeric films.

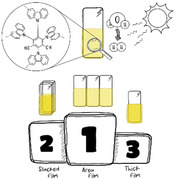

## 1 Introduction

Hydrogen is a zero‐emission fuel and an energy carrier with high energy density. The annual production of hydrogen reached 95 Mt in 2022, dominated by natural gas reforming as main production pathway.^[^ ^1^, ^2^ ^]^ With the average of 11 kg_CO2eq_/kg_H2_ large amounts of CO_2_ are emitted during direct methane reforming.^[^ ^2^ ^]^ In contrast, electrochemical water splitting using renewable energy sources is considered to produce low‐emission hydrogen with just 2.02 kg_CO2eq_/kg_H2_.^[^ ^2^, ^3^ ^]^ Firstly, sunlight or wind is converted to electrical energy which is then used to power an electrolyzer splitting water electrochemically at temperatures above 50 °C.^[^ ^4^, ^5^ ^]^ Compared to water electrolysis photocatalytic water splitting is a direct pathway to produce green hydrogen and oxygen using sunlight under ambient conditions.^[^ ^6^ ^]^ In past decades mainly inorganic semiconductors were studied and optimized for this application.^[^ ^7^, ^8^, ^9^, ^10^ ^]^ Within the last decade, however, conjugated microporous polymers (CMPs) also proved to be promising photocatalysts for hydrogen production and water splitting.^[^ ^11^, ^12^, ^13^, ^14^, ^15^, ^16^ ^]^

This is mainly due to their extended π‐conjugated systems, whose optoelectronic properties can be tuned by the choice and combination of monomers. In addition, due to the covalent linkages between the shape‐persistent monomers, CMPs feature a good stability and a high specific surface area.^[^ ^17^ ^]^ Monomers carrying several carbazole groups can easily be converted into carbazole‐based porous organic polymers (C‐POPs) by oxidative polymerization. This process is cost‐effective since cheap oxidants like iron (III) chloride at ambient temperature are applied. Due to their nitrogen‐rich backbone C‐POPs are mainly known for their high gas adsorption properties and as macroligands in heterogeneous catalysis.^[^ ^18^, ^19^, ^20^ ^]^ Recently, C‐POPs also gained attraction in photocatalysis.^[^ ^20^, ^21^, ^22^, ^23^, ^24^, ^25^ ^]^ Especially, donor‐acceptor based 1,2,3,5‐tetrakis(carbazol‐9‐yl)‐4,6‐dicyanobenzene (4CzIPN) C‐POPs were thoroughly investigated due to their photophysical properties like thermally activated delayed fluorescence (TADF) in photocatalysis.^[^ ^26^, ^27^, ^28^, ^29^, ^30^, ^31^ ^]^ Su and co‐workers studied the substitution positions and numbers of oxidative polymerized C‐POPs containing carbazole as donor and nitrile groups as acceptor. In sacrificial photocatalytic hydrogen evolution reaction using the oxidative polymerized 4CzIPN, an H_2_ evolution rate of 1.3 mmol g^−1^ h^−1^ (λ > 420 nm) was achieved.^[^ ^26^ ^]^ However, despite the straightforward synthesis of powder‐based photocatalysts, upscaling of the photocatalytic process to produce industrial relevant amounts of hydrogen is challenging when a powder dispersion should be used.^[^ ^32^, ^33^ ^]^ This includes a decrease in activity per unit mass when higher amounts of photocatalyst are applied, attributed to increased light scattering and limitations of the reaction vessels using powdery photocatalysts.^[^ ^34^, ^35^, ^36^ ^]^ In contrast, photocatalysts fabricated as films proved to be easily upscaled and recovered, and possess a higher activity compared to their powder counterparts.^[^ ^34^, ^35^, ^37^ ^]^ Cooper and co‐workers prepared a non‐porous dibenzo[b,d]thiophene sulfone polymer with tri(ethylene glycol) sidechains which evolves roughly 5 times the amount of hydrogen when applied as a film (13.9 mmol g^−1^ h^−1^) compared to powder dispersion (2.9 mmol g^−1^ h^−1^). While thicker films did not improve the catalytic activity significantly, an increase in activity was observed by stacking the film covered slides.^[^ ^38^ ^]^ Gopinath and co‐workers reported that thin films of titania produced 3 times more hydrogen in photocatalysis compared to the powder state.^[^ ^36^ ^]^ In fact, shaping materials into films, membranes or other macroscopic objects got more research interest in photocatalytic water splitting and other applications within the last years.^[^ ^39^, ^40^, ^41^, ^42^, ^43^, ^44^, ^45^, ^46^, ^47^, ^48^, ^49^, ^50^, ^51^ ^]^

Herein, oxidative polymerization is performed electrochemically to prepare thin films of C‐POPs.^[^ ^52^ ^]^ Through multicycle cyclic voltammetry (CV) the polymerization is performed in a two‐electron oxidation process on a transparent fluorine‐doped tin oxide (FTO) electrode, controlling the film thickness by the number of performed cycles.^[^ ^53^ ^]^ Till now, only a few studies focussed on the photocatalytic activity of such polymerized C‐POP‐based thin films. One example is shown by Bergens and co‐workers who investigated the thickness dependence of 1‐imidazole‐2,4,6tri(carbazol‐9‐yl)‐3,5‐dicyanobenzene (3CzImIPN) films in the photocatalytic stilbene isomerization.^[^ ^54^ ^]^

**Figure 1:**
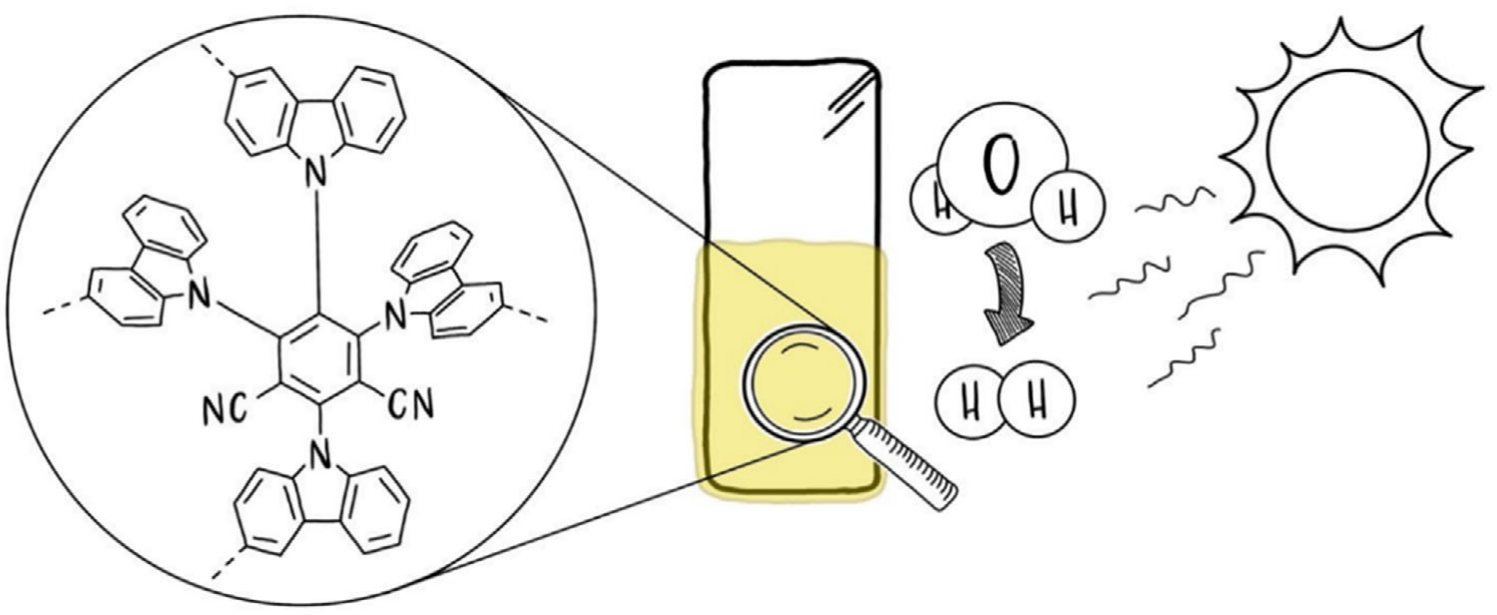
Schematic representation of 4CzIPN‐based polymeric film in photocatalytic hydrogen evolution reaction.

To the best of our knowledge, we present here for the first time the electrochemical polymerization, the photocatalysis, and the photolithographic micro structuring of C‐POP‐based polymeric films in photocatalytic hydrogen evolution (**Figure** **1**).

## 2 Results and Discussion

### 2.1 Polymer Synthesis and Characterization

The porous polymer of 4CzIPN was synthesized in bulk as reference material by oxidative polymerization with iron(III)‐chloride as oxidizing agent in anhydrous chloroform at room temperature using a modified procedure compared to previously reported conditions (see SI for details).^[^ ^26^ ^]^

The permanent microporosity of the bulk polymer was studied by nitrogen sorption measurements at 77 K (Figure S1a, Supporting Information). A typical type I isotherm was observed with a calculated Brunauer–Emmett–Teller (BET) surface area of 979 m^2^ g^−1^ exceeding the reported surface area of 649 m^2^ g^−1^.

The chemical composition was confirmed by Fourier transform infrared (FT‐IR) spectroscopy, ^13^C cross polarization magic angle spinning (CP‐MAS) NMR spectroscopy, and X‐ray photoelectron spectroscopy (XPS). The signal at 2236 cm^−1^ in the FT‐IR spectrum (Figure S1b, Supporting Information) correspond to the C‐N stretching mode of the nitrile group, which remains unaffected during the course of polymerization reaction and work‐up procedure of the bulk polymer. Additionally, the presence of the nitrile groups was confirmed with a signal at 111 ppm in the ^13^C CP‐MAS NMR spectrum (Figure S1c, Supporting Information).^[^ ^27^, ^30^, ^55^ ^]^ Additionally two distinct signals were observed at 125 and 139 ppm, belonging to the aryl carbons and carbons next to the carbazolyl nitrogen atom, respectively.^[^ ^21^, ^24^, ^30^ ^]^ In the deconvoluted nitrogen 1s core level XPS spectrum (Figure S2, Supporting Information) two distinct peaks were found at 400.7 and 399.6 eV corresponding to the carbazole nitrogen and the nitrile, respectively.^[^ ^21^, ^24^ ^]^

**Figure 2:**
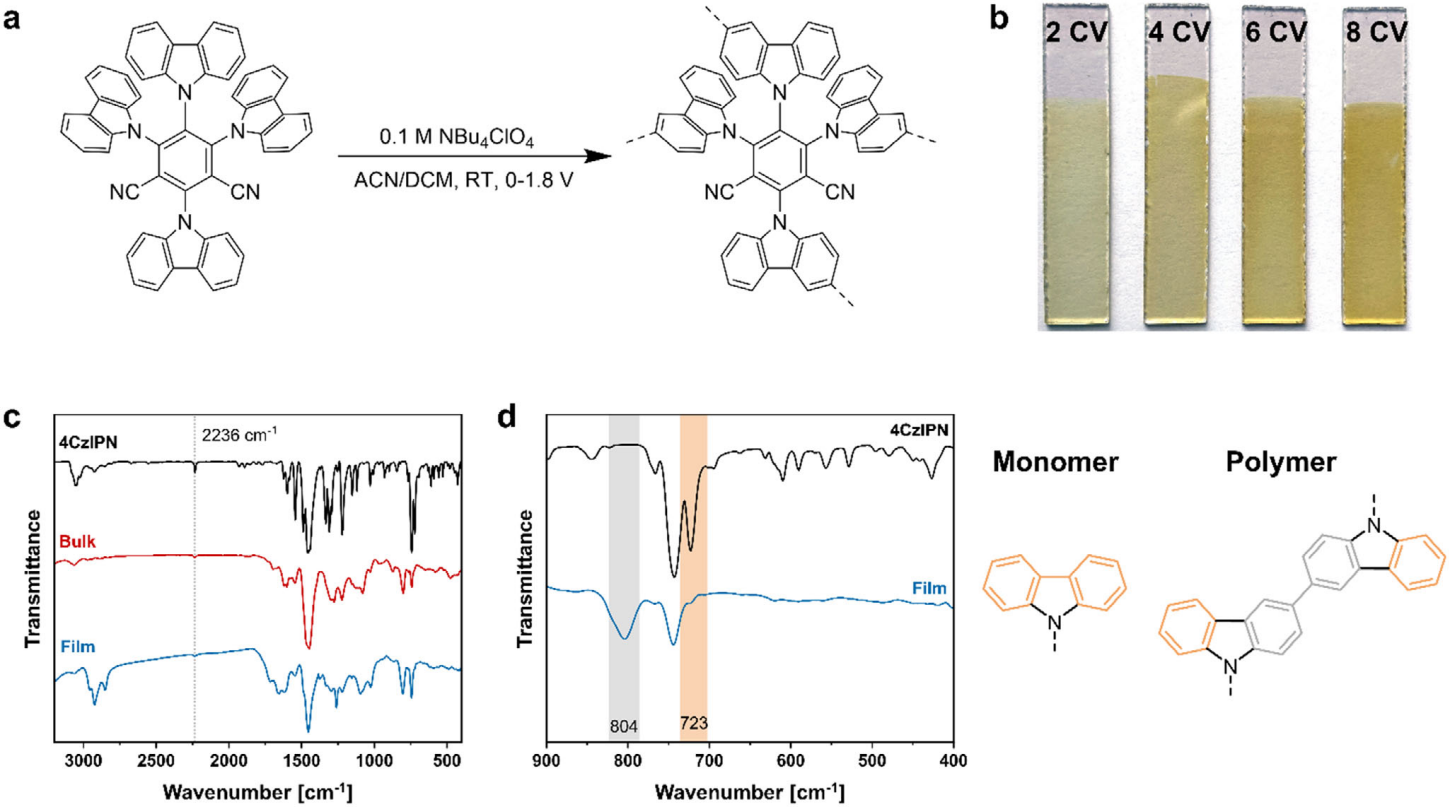
Synthetic protocol of electro‐polymerization of 4CzIPN (a). Images of the obtained films after 2, 4, 6, and 8 CV cycles (b). FT‐IR spectra of the as synthesized film, compared to the monomer and bulk polymer (c) and the enlarged fingerprint area of the film in comparison to 4CzIPN monomer (d).

The synthesis of the polymer films (**Figure** **2**a) was performed in a three‐electrode set‐up using a FTO‐coated glass slide as working electrode, a platinum wire as counter electrode, and an Ag^0^/AgCl (3 M NaCl) electrode as reference electrode. The electro‐polymerization of the 1.0 mM 4CzIPN solution was accomplished using cyclic voltammetry (CV) from 0 to 1.8 V (scan rate: 50 mV s^−1^). Film thickness was varied during CV polymerization by performing 2, 4, 6, and 8 CV cycles per sample on an electrode area of ≈3 cm^−2^ (Figure 2b). Above 10 CV cycles the film started to peel off the electrode either during washing or drying process, which did not allow further photocatalytic studies. With each cycle the oxidation peak of the carbazole moiety shifted continuously from 1.0 V vs Ag^0^/AgCl (first CV cycle) toward 1.4 V vs Ag^0^/AgCl (after 8 CV cycles) due to the polymerization of carbazole functionalities (Figure S3, Supporting Information).^[^ ^54^ ^]^ This upshift of the oxidation of carbazole peaks indicates a continuous growth of the film.^[^ ^56^, ^57^ ^]^

**Figure 3:**
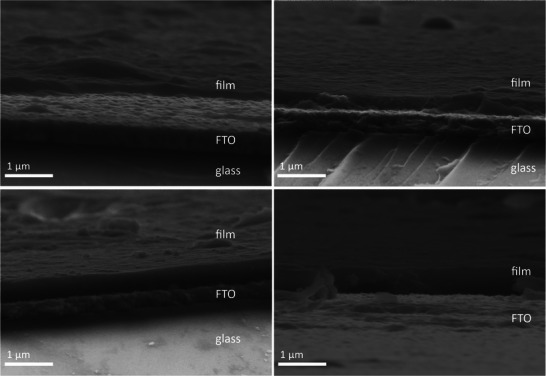
FESEM images of the cracked film on FTO electrodes after 2 (a), 4 (b), 6 (c), and 8 CV cycles (d).

As it was already proven for the bulk polymer after oxidative polymerization, the nitrile peak at 2236 cm^−1^ of 4CzIPN was present in FT‐IR spectroscopy after electro‐polymerization (Figure 2c). In the fingerprint region (Figure 2d) the prominent peak at 723 cm^−1^, corresponding to disubstituted phenyl rings of the carbazolyl moiety in the 4CzIPN monomer, nearly disappeared in the bulk and film polymer, and a new C‐H vibration band at 804 cm^−1^ appeared, corresponding to trisubstituted phenyl rings due to polymerization.^[^ ^58^, ^59^, ^60^ ^]^ The high resolution deconvoluted N 1s core level XPS spectra of each film (Figure S4, Supporting Information) further confirmed the chemical structure with two distinct peaks at 400.7 and 399.6 eV confirming the carbazole and nitrile nitrogen, respectively. Field emission scanning electron microscopy (FESEM) analysis showed the formation of a homogeneous film on the FTO electrode. The thickness of each film was determined at the cross‐section after cracking each film (**Figure** **3**). The film grows from 190 nm (2 CV cycles) to 390 nm (4 CV cycles) and further to 490 nm (after 6 CV cycles) and 570 nm (after 8 CV cycles), showing that the amount of formed film within each cycle decreased with further CV cycles.

The optical bandgap and the band positions of a photocatalyst are crucial for its performance, therefore the properties of the bulk and film polymer were investigated by diffuse reflectance UV‐Vis (DR‐UV‐Vis) spectroscopy. The bulk polymer appears as a slightly orange colored powder and showed an absorption edge at around 500 nm (Figure S5, Supporting Information) with an optical bandgap of 2.2 eV, calculated from the tauc plot (Figure S6, Supporting Information). In contrast, the films showed a first absorption step at 480 nm and an absorption edge at 440 nm (Figure S5, Supporting Information). The band gap was determined by tauc plot to be at 2.57 ± 0.02 eV (Figure S6, Supporting Information).^[^ ^61^ ^]^ Along with the optical band gap, the band positions are crucial to determine the oxidation or reduction potential of the photocatalyst after illumination. Therefore, valence band XPS (VB XPS) was conducted to determine the valence band position (Figure S7 and Table S1, Supporting Information). The VB positions were calculated to be at −5.64 eV vs vacuum level for the bulk polymer and at −5.72 ± 0.09 eV vs vacuum level for the polymeric film. The conduction band (CB) positions are calculated by adding the optical bandgap with the VB and are in good agreement with the electrochemical determined ones (Figure S8 and Table S1, Supporting Information).^[^ ^54^ ^]^ Based on the resulting band positions, the polymer should be able to reduce protons to hydrogen, since the CB is energetically above the HER potential of −4.44 eV vs vacuum. In addition, the VB level is below the oxidation potential of ascorbic acid (AA) and triethanolamine (TEOA), which fulfill the requirements for both sacrificial agents (SA) to be applied in photocatalytic hydrogen evolution reaction (HER).^[^ ^62^ ^]^ Compared to the bulk polymer, the VB position of the films is below the oxygen evolution reaction potential of −5.67 eV vs vacuum, showing the possibility to be applied in overall water splitting. Besides the VB and CB level the wettability of the polymer surface has a huge impact on the photocatalytic activity. Therefore, the contact angle of water on the film surface after 2 CV cycles was measured and the polymer proved to be rather hydrophilic and wettable with a contact angle θ_c_ of 77.7° ± 2.8° (Figure S9 and Table S2, Supporting Information). To understand the materials electronic behavior under illumination, electrochemical impedance spectroscopy (EIS) was conducted of the film after 2 CV cycles to study the interfacial charge transfer resistance. Under illumination a smaller semicircle was observed in the Nyquist plot (Figure S10a, Supporting Information) indicating a decrease in the charge transfer resistance due to illumination. For the thin film after 2 CV cycles and the thick film after 8 CV cycles the photocurrent response was measured (Figure S10b,c, Supporting Information). Upon irradiation an anodic photocurrent was observed, confirming the n‐type semiconductor character of the polymeric film.^[^ ^63^ ^]^

### 2.2 Photocatalysis

The photocatalytic activity of the bulk polymer for HER in aqueous solution was investigated under visible light irradiation (λ > 400 nm, 400 nm UV‐cutoff filter, 300 W xenon lamp, 15 cm distance, intensity 1.6 W cm^−2^) using 2.4 wt.% Pt as co‐catalyst and either AA (pH = 2.6) or TEOA (pH = 10.7) as sacrificial agent (SA). After 2 h of illumination, a linear fit was applied and the activity was calculated as 1.0 mmol g^−1^ h^−1^ for AA and 4.6 mmol g^−1^ h^−1^ for TEOA (Figure S11, Supporting Information), respectively. Without the addition of SA no hydrogen evolution was observed, as expected since the VB is above the OER potential of −5.67 eV vs vacuum. The absence of Pt as co‐catalyst resulted in an activity of 165 µmol g^−1^ h^−1^ using TEOA as SA, which is roughly 30 times lower compared to the 4.6 mmol g^−1^ h^−1^ produced with additional co‐catalyst (Figure S12, Supporting Information). Applying the most active conditions, TEOA as SA and Pt as co‐catalyst, the apparent quantum efficiencies (AQEs) were calculated to be 2.7% at 365 nm.

**Figure 4:**
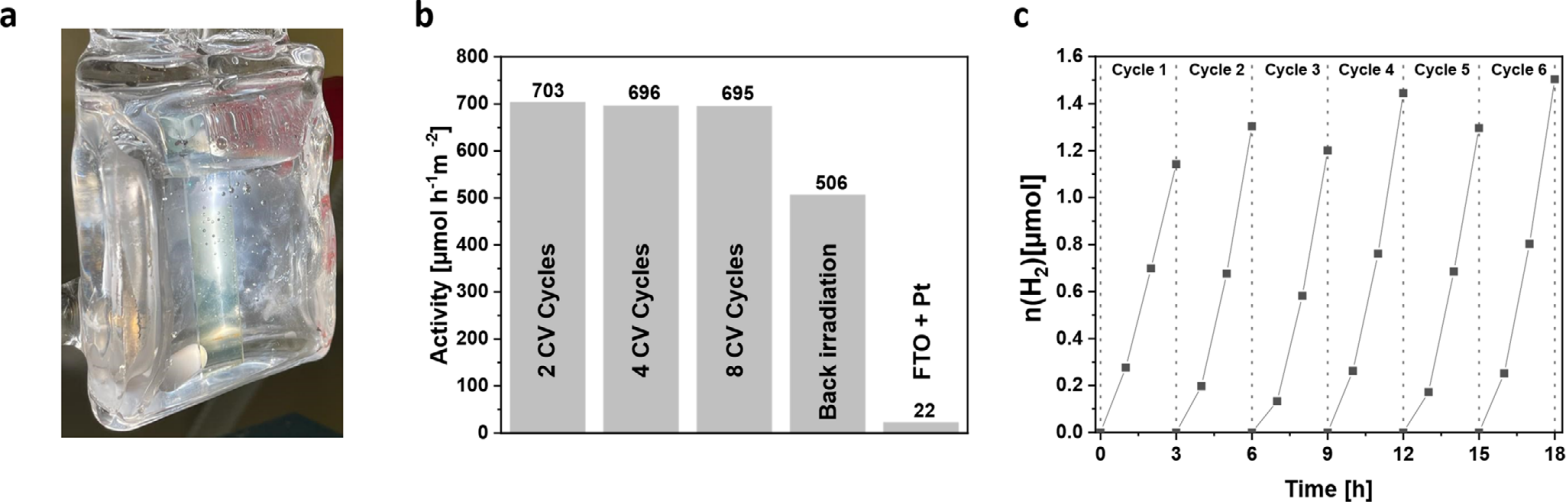
Image of the 2 CV cycles film illuminated with light in the photoreactor (a). Comparison of the HER performance of the films compared to back irradiated film and blank experiment without photocatalyst under visible light (> 400 nm) in water/triethanolamine mixture (4:1) and Pt as co‐catalyst (b). Six cycles of hydrogen evolution of the 2 CV cycles film (film area: 2.8 cm^2^) (c).

Based on the results of the bulk polymer, the photocatalytic studies for the films were performed using TEOA as sacrificial agent and Pt as co‐catalyst at pH = 10.7. The films were placed vertically in the photoreactor and illuminated from the front side (**Figure** **4**a). The activity (calculated over all 5 points) of the films were almost similar independent on the film thickness with 703 µmol h^−1^ m^−2^ (2 CV cycles, Figure S13, Supporting Information), 696 µmol h^−1^ m^−2^ (4 CV cycles, Figure S14, Supporting Information) and 695 µmol h^−1^ m^−2^ (8 CV cycles, Figure S15, Supporting Information). Irradiation from the back of the FTO electrode results in a decrease in activity to 506 µmol h^−1^ m^−2^ for the film after 8 CV cycles (Figure S16, Supporting Information) probably due to partial absorption and reflection of the incident light on the glass/FTO/polymer film. Compared to the activity of the films (Figure 4b), the amount of produced hydrogen with the bare FTO electrode is negligible (22 µmol h^−1^ m^−2^, Figure S16, Supporting Information). The catalysis was reproducible over different batches (Figure S17, Supporting Information) showing an activity of 702 µmol h^−1^ m^−2^ (Batch 1), 698 µmol h^−1^ m^−2^ (Batch 2) and 705 µmol h^−1^ m^−2^ (Batch 3). The addition of less and more Pt co‐catalyst led to a decrease in photocatalytic activity (Figure S18, Supporting Information). The AQY of the thin film was calculated to be 0.3% at 365 nm.

Additionally, the recyclability was studied with the thin film (2 CV cycles) by replacing the solution after each cycle and degassing with argon for 20 min before starting irradiation for 3 h. After the initial addition and photo‐deposition of the Pt co‐catalyst in the first catalytic cycle, no co‐catalyst was added in further cycles. The films showed a stable photocatalytic production of hydrogen for at least 6 cycles indicating its excellent recyclability (Figure 4c). XPS analysis done after the cycling studies confirmed the chemical stability of the film during photocatalysis (Figure S19, Supporting Information). After 6 photocatalytic cycles, only two nitrogen species, the carbazolyl nitrogen at 400.7 eV and the nitrile nitrogen at 399.7 eV, were observed without a signal corresponding to oxidized nitrogen species. Additionally, in the core‐level spectra of Pt a doublet at 71.2 and 74.5 eV corresponding to 4f 3/2 and 4f 1/2 orbital of metallic platinum confirmed the successful immobilization of Pt co‐catalyst at the films surface.

Transmission electron microscopy analysis after catalysis revealed the presence of crystalline Pt nanoparticles and clusters distributed over the whole polymeric film surface (Figure S20, Supporting Information). Additionally, a shoulder in the absorption edge at 462 nm in the DR‐UV‐Vis spectrum could be observed due to the photo‐deposited co‐catalyst (Figure S21, Supporting Information).

**Figure 5:**
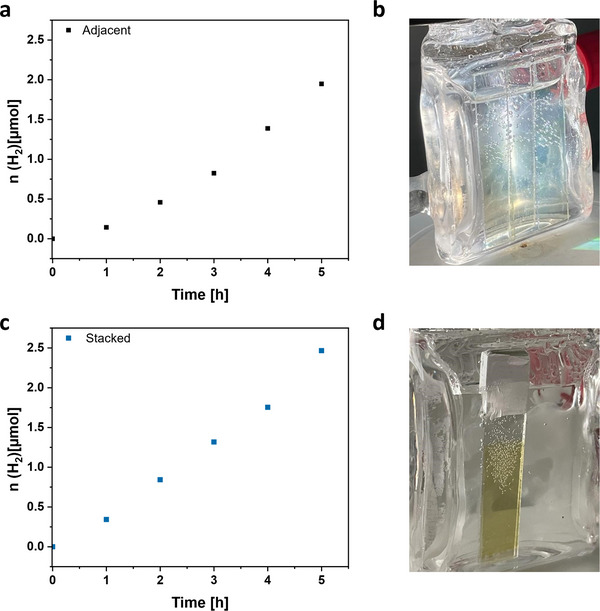
Time course of H_2_ production of three 2 CV cycles films placed adjacent to each other (a) and image during illumination under visible light (>400 nm) in a water/triethanolamine mixture (4:1) and Pt as co‐catalyst (b). Time course of H_2_ production of the same films stacked (c) and images of the stacked films under visible light (>400 nm) in a water/triethanolamine mixture (4:1) (d).

The observation that the activity of the films was independent on the film thickness indicate that the photocatalysis is limited by mass transfer and mainly occurs on the outer film surface and not within the bulk of the microporous polymer film. At least considering the film thickness, it seems unlikely that a catalytic reaction is possible in the film at points deeper than 190 nm. To illustrate this, three films (2 CV cycles) were placed next to each other in the reactor. Based on the film thickness, this represents the same amount of polymer as obtained after 8 CV cycles, but with the accessible outer surface being three times greater. Compared to a single film the photo‐deposition of Pt required more time, therefore the catalytic activity was calculated with a linear fit after 3 h (**Figure** **5**a,b). The activity of 0.50 µmol h^−1^ is 2.6 times the activity of one thick film (8 CV cycles, 0.19 µmol h^−1^). Even when the same three films were stacked back‐to‐back instead of placing them adjacent, an activity of 0.48 µmol h^−1^ is observed, which is 2.5 times the activity of the thick film after 8 CV cycles (**Figure** 5c,d). Notably, such stacking would not increase the illuminated area of a potential device compared to a single film, while the hydrogen content obtained is significantly increased, which is of high interest for practical solutions. In both cases the increase of the external film area led to an increase in activity, validating that the photocatalytic reactions mainly occur on the polymeric films′ outer surface.

**Figure 6:**
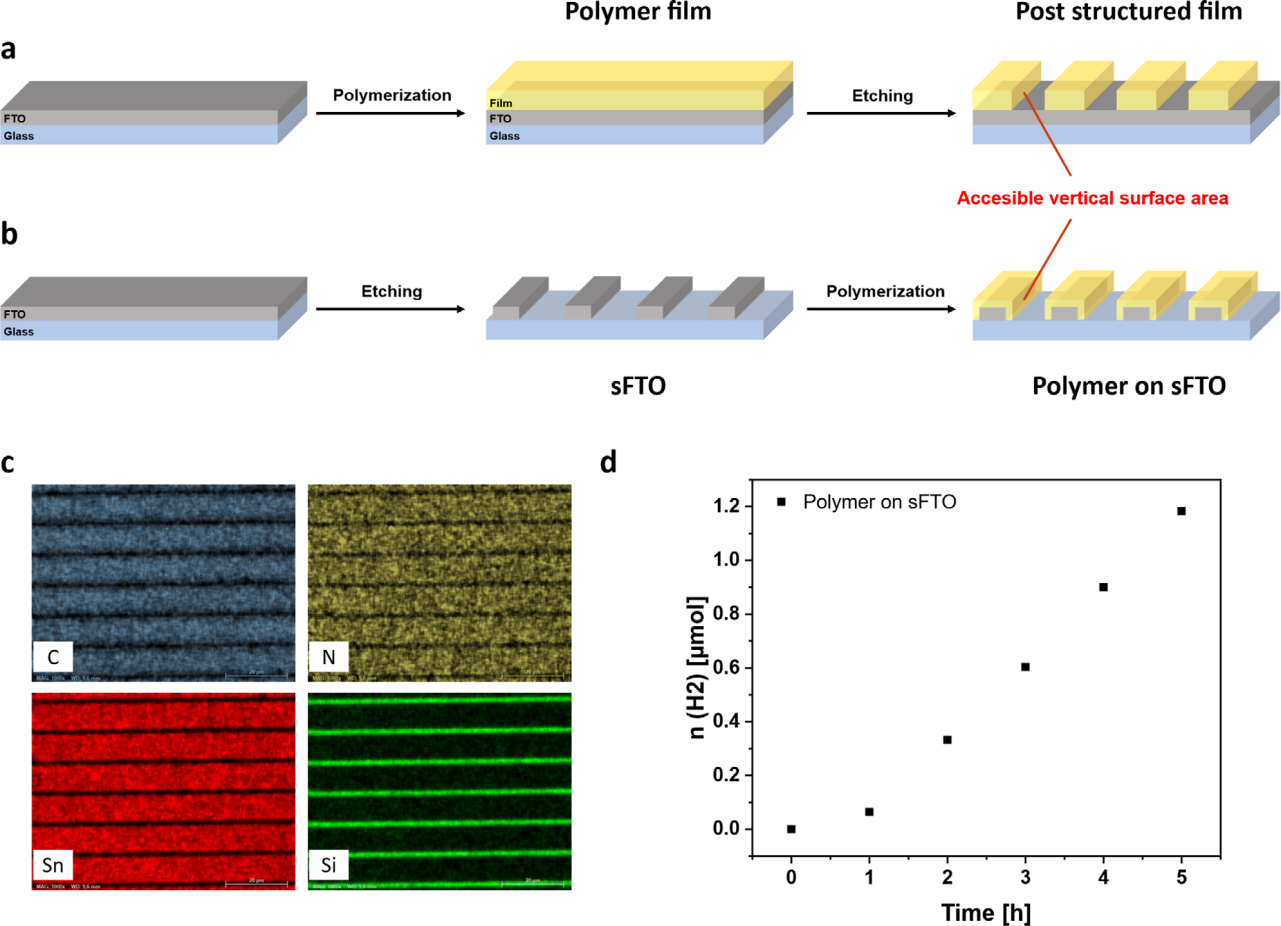
Scheme of post‐synthetic structuring the 4CzIPN film after 8 CV cycles via laser ablation (a). Scheme of film synthesis onto a laser‐ablated microstructured FTO electrode (b). SEM‐EDX of the polymer film on a microstructured FTO electrode (c). Time course of H_2_ production of the polymer film on sFTO electrode (film area: 4 cm^2^) under visible light (> 400 nm) in a water/triethanolamine mixture (4:1) and Pt as co‐catalyst (d).

Instead of increasing the outer surface area by stacking or placing several thin films next to each other, another solution might be utilization of microstructured polymers. Although it can be assumed that the micropores (i.e. <2 nm) in the film are too small to allow rapid transfer of water molecules or SAs into the bulk of the film, this will certainly be different for defined voids in the scale of micrometers, which can be introduced into the film by various lithographic techniques. By applying photolithography methods, a polymer film or a FTO electrode can be directly structured.^[^ ^64^, ^65^, ^66^ ^]^ First, microstructuring of the polymer film via laser ablation was performed after synthesis. Successful line patterning of the film after 8 CV cycles by removing stripes of 1 µm polymer at 2 µm intervals over the entire surface of the film (**Figure** **6**a) was demonstrated by SEM‐EDX analysis (Figure S22, Supporting Information). Nonetheless XPS analysis revealed a chemical change of the polymer structure (Figure S23a, Supporting Information). In the deconvoluted high resolution N1s XPS spectrum an additional nitrogen species at 403.0 eV is present, not observed in the pristine polymers, which is ascribed to oxidized nitrogen species. This partial oxidation of the polymer film led to a reduction in the photocatalytic HER to 421 µmol h^−1^ m^−2^ (calculated over the last 5 points, Figure S24, Supporting Information). Compared to post‐synthetic etching of the polymer film, prior microstructuring of the FTO electrode enables the direct growth of a microstructured polymer film, whereby both the outer surface area is increased and less polymer is used (Figure 6b). To ensure sufficient conductivity, half of the electrode was line patterned every 10 µm and a thick polymer film with 8 CV cycles was synthesized to achieve a high vertically accessible surface area (Figures S25 and S26, Supporting Information). The XPS analysis revealed the same chemical species compared with the unstructured film (Figure S23b, Supporting Information). The SEM analysis showed the successful synthesis of a polymer film with stripes of 8.2 µm polymer width interrupted by lines with a width of 1.8 µm (Figure S27, Supporting Information). The SEM‐EDX analysis proved the etching of FTO (sFTO) and the polymer formation on the remaining sFTO areas (Figure 6c). This modification led to the use of 18% less polymer amount within the modified area, which is a decrease of 9% for the whole polymer film since just half of the electrode was modified via laser ablation. Due to the microstructuring, the vertical surface is now accessible which results in a similar outer surface area compared to an unstructured polymer film. Even though a lower amount of polymer is present on the sFTO, the photocatalytic activity was maintained with 701 µmol h^−1^ m^−2^ (calculated over the last 5 points, Figure 6d). It is expected that by varying the film thickness and the structuring pattern of the FTO electrode, the accessible outer surface of the polymer film can be further increased with minimal use of material, which could lead to space‐ and cost‐saving devices for practical photocatalytic applications.

## 3 Conclusion

In summary, a 4CzIPN bulk and film polymer was synthesized via oxidative and electro‐polymerization and the photocatalytic HER activity of the powder and the film was studied. With increasing film thickness the same photocatalytic activity was observed. However, using the same mass of films divided into several thinner films the activity was increased. This indicates that the catalysis mainly occurs on the films outer surface rather than in the micropores due to mass transport limitations. Instead of synthesizing one thick film, it is therefore beneficial to spread the same amount of polymer on a larger electrode area or to stack several thin films of the same size to increase the activity. Additionally, microstructuring an electrode via laser ablation results in an increase of the accessible surface area and the activity together with using less material. Furthermore, the films can be easily recycled without the use of any additional co‐catalyst after deposition.

## Conflict of Interest

The authors declare no conflict of interest.

## Author Contributions

V.D. conceived the project, performed the experiments, and wrote the manuscript with the support of S.G., G.M., J.S., and A.T. L.G. and A.L. recorded solid‐state ^13^C CP‐MAS NMR data. FESEM measurements were done by I. K. Photocatalysis was performed together by V.D., S.G., and H.K. IR spectroscopy, and contact angle measurements were done by G.M. The laser ablation experiments were performed by E.B. and D.O.

## Supporting information



Supporting Information

## Data Availability

The data that support the findings of this study are available from the corresponding author upon reasonable request.
