# Supplementary material for: Carbazole‐Based Thin Microporous Polymer Films for Photocatalytic Hydrogen Evolution
Source: Adv Mater. 2025 Jun 23;37(38):2506689. doi: 10.1002/adma.202506689 (PMC12464628; doi:10.1002/adma.202506689)
Supplement: Supplementary file 1 — Supporting Information [file ADMA-37-2506689-s001.pdf]

ADVANCED MATERIALS

Supporting Information

for *Adv. Mater.*, DOI 10.1002/adma.202506689

Carbazole-Based Thin Microporous Polymer Films for Photocatalytic Hydrogen Evolution

*Veit Dippold, Hüseyin Küçükkeçeci, Eugenia Bosler, Johannes Schmidt, Samrat Ghosh, Gregor Michl, Islam E. Khalil, Lisa Gerland, Adam Lange, Dirk Oberschmidt and Arne Thomas**

Supporting information

Carbazole-Based Thin Microporous Polymer Films for Photocatalytic Hydrogen Evolution

Veit Dippold[a], Hüseyin Küçükkeçeci[a], Eugenia Bosler[c], Johannes Schmidt[a], Samrat Ghosh[a], Gregor Michl[a], Islam E. Khalil[a], Lisa Gerland[b], Adam Lange[b], Dirk Oberschmidt[c] and Arne Thomas[a]

[a]Department of Chemistry/Functional Materials, Technische Universität Berlin, Berlin, Germany.

[b]Research Unit Molecular Biophysics, Leibniz-Forschungsinstitut für Molekulare Pharmakologie, Berlin, Germany.

[c] Department of Micro and Precision Devices, Technische Universität Berlin, Berlin, Germany.

I. Experimental

I.I Materials

All reagents were used without further purification. 4CzIPN was purchased from BLDpharm. Dichloromethane and ethanol were bought from Carl Roth and acetonitrile from Fisher Scientific. Tetra-n-butylammonium perchlorate and Tetra-n-butylammoniumhexafluorophosphat were purchased from Thermo Scientific. FTO coated glass electrodes were purchased from MSE supplies (Size: 100 x 100 x 1.1 mm, resistance: 15 Ohm/m^2) and cut to the size 10 x 50 mm.

I.II Characterization

Low pressure nitrogen sorption experiments were conducted at 77 K up using an Quadrasorb SI from Quantachrome equipped with a Quantachrome CyroCooler for temperature regulation. The isotherms were evaluated by "ASiQwinTM" from Quantachrome Instruments. The calculated Brunnauer-Teller-Emmet (BET) surface area of the bulk polymer was determined by measuring the nitrogen sorption isotherms at 77 K.

The solid-state NMR-measurements were performed on a Bruker Avance 400 MHz spectrometer equipped for cross-polarization magic angle spinning (CP-MAS). The ^{13}C CP-MAS spectra were measured at 100 MHz at a spinning rate of 10 kHz. All spectra were evaluated using Bruker´s software "TopSpin".

Solid-state Diffuse Reflectance Ultraviolet-visible Spectroscopy (UV-Vis) spectra were recorded on a Varian Cary 300 UV-Vis spectrophotometer. The bulk polymer was distributed on the sample holder. The film was placed in the sample holder and the background, a blank FTO electrode, was subtracted.

The Thermo Scientific K-AlphaTM + X-ray Photoelectron Spectrometer System with a hemisphere 180° dual-focus analyzer with a 128-channel detector was used to perform X-Ray Photoelectron Spectroscopy (XPS) measurements. The monochromator used micro-focused Al-K-alpha irradiation. The spectra were evaluated with Thermo Scientific Software "Avantage". Before the deconvolution, the binding energy positions were calibrated with C 1s (284.8 eV).

The film synthesis and electrochemical analysis was performed at a Gamry Reference 600 Potentiostat at room temperature. A FTO coated glass slide was used as working electrode, a Pt wire as counter electrode and an Ag^0/AgCl (3 M NaCl) as reference electrode, respectively. The reference electrode was calibrated with ferrocene (0.2 M) in a degassed solution of 0.1 M $TBAPF_6$ in acetonitrile. The electrochemical impedance spectroscopy (EIS) and transient photocurrent (PC) were conducted in 0.2 M Na_2SO_4 solution in water. A Lumatec Superlite device (400-700 nm) was used to illuminate the films from the back.

Field emission scanning electron microscopy (FESEM) analysis was done at a ZEISS GeminiSEM 500. To determine the film thickness, the electrodes covered with film were broken and the breaking edge was directly studied without additional gold coating in nanoVP mode.

Transmission electron microscopy (TEM) analysis was performed at a Tecnai G2 20s-Twin. For the measurement, the film after catalysis was scratched of the electrode onto the copper grid.

The transmission Fourier-Transform infrared (FT-IR) spectra were recorded on a Bruker Vertex70V equipped with a DLATGS detector (4 cm^{-1} resolution). For the KBr pellets 300 – 400 µg of the bulk

polymer or the scratched off film was mixed with 230 mg of KBr. The measurement was referenced against a blank KBr pellet.

The contact angle was measured with a Krüss FM40 EasyDrop device. A drop of 3 µL Milli-Q water was applied on the film surface and the contact angle was measured using the conic section method.

II. Experimental Procedures

II.I Bulk Polymer

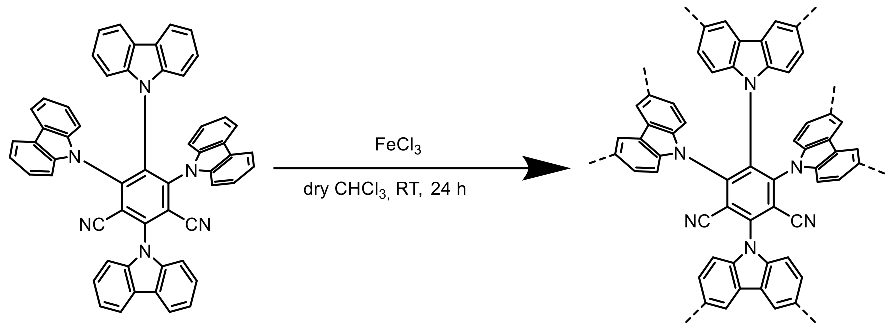

Under argon atmosphere 658 mg anhydrous iron (III) chloride (4.05 mmol, 32 eq.) was placed in an oven dried 500 mL Schlenk flask equipped with a dropping funnel and dispersed in 60 mL anhydrous chloroform. Afterwards, 100 mg 4CzIPN (0.13 mmol, 1eq.) dissolved in 50 mL anhydrous chloroform was added dropwise with a dropping funnel at room temperature. After complete addition and washing with further 10 mL chloroform the mixture was stirred at room temperature under argon atmosphere. After 24 hours the polymerization was quenched with 100 mL methanol and filtered. After soxleth extraction with methanol for three days to remove remaining iron, the polymer was dried at 80 °C under vacuum yielding in 91 mg of an orange polymer.

II.II Polymer film

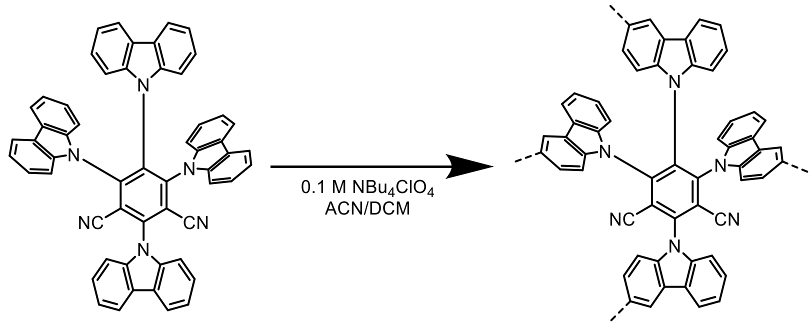

For the electrochemical polymerization 4CzIPN (39.4 mg, 0.05 mmol, 1.0 mM) and tetrabutylammonium perchlorate (1.4 g, 5.0 mmol, 0.1 M) were dissolved in a mixture of dichloromethane (40 mL) and acetonitrile (10 mL). The solution was placed in a three-electrode cell and degassed with nitrogen for 10 min. A FTO covered glass slide was applied as working electrode, platinum wire and Ag^0/AgCl (3 M NaOH) were used as counter and reference electrode. The films were

synthesized via cyclic voltammetry by scanning from 0.2 V to 1.8 V with a scan rate of 50 mV s^{-1}. Films with different thicknesses on an area of 3 cm^{-2} were synthesized by varying the amount of performed CV cycles. After successful electro-polymerization a potential of 0 V was applied for 120 s to discharge the material. To remove remaining electrolyte salt, the films were washed carefully with water and ethanol several times before drying at room temperature.

II.III Micro-structuring via laser ablation

The micro-structuring via laser ablation was performed on a WOP FemtoLAB device. The polymer film and the FTO electrode were line structured with a 515 nm laser pulse (objective: 50x NA 0.42 (Mitutoyo), speed: 15 mm/s, pulse duration: 244 fs, frequency: 601.8 kHz, pulse density: 50000/mm) every 10 µm. The polymer film was etched with a pulse power of 253.35 mW and the FTO electrode was structured with 897.75 mW.

III. Characterization data

III.I Bulk Polymer

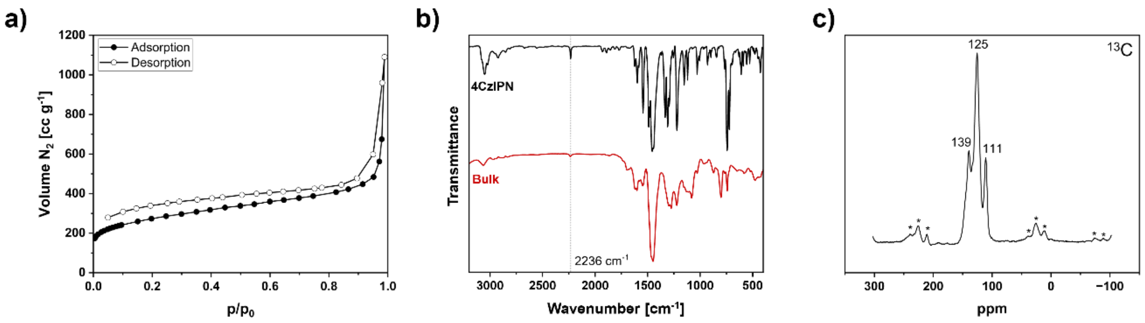

Figure S1: Nitrogen sorption isotherms at 77 K of the bulk polymer (a). Normalized FT-IR spectrum of bulk polymer in comparison to the 4CzIPN monomer (b). Solid-state ^{13}C CP-MAS NMR spectrum (c). Spinning side bands are marked with asterisks.

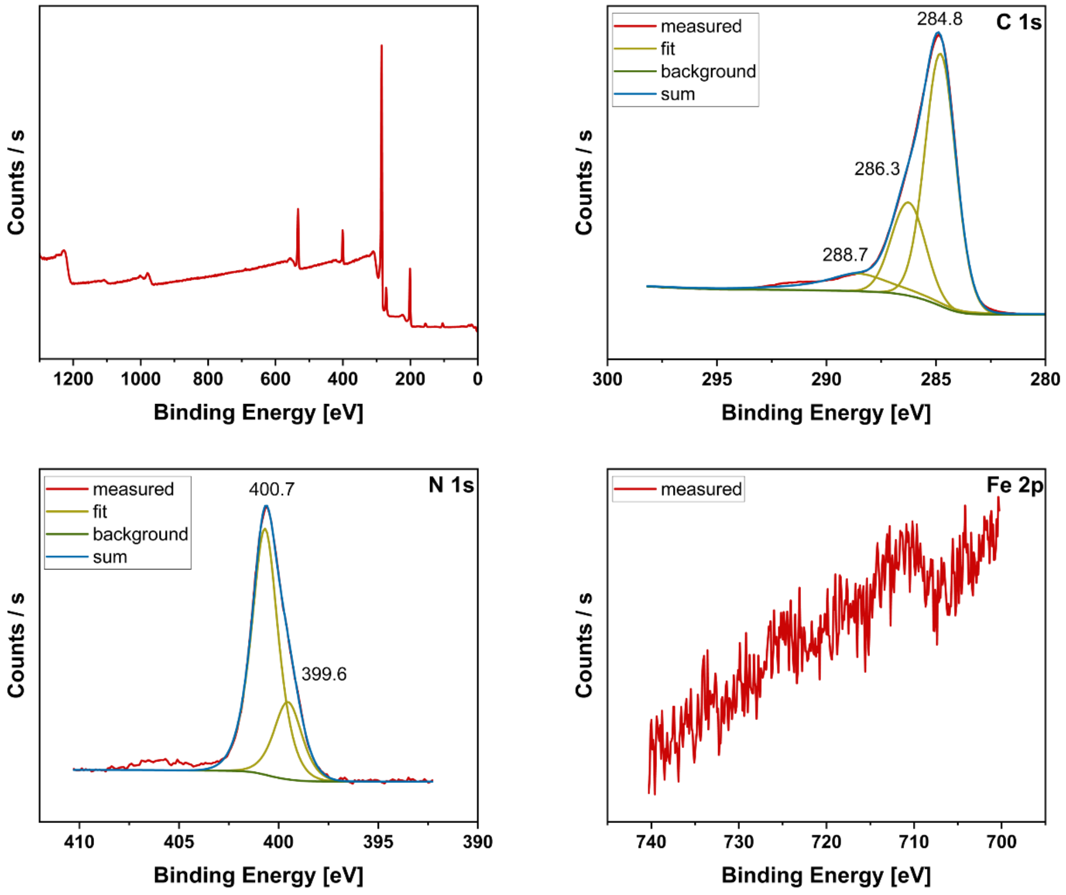

Figure S2: Survey, C 1s, N1s and Fe 2p XPS spectra of bulk polymer.

III.II Polymer Film

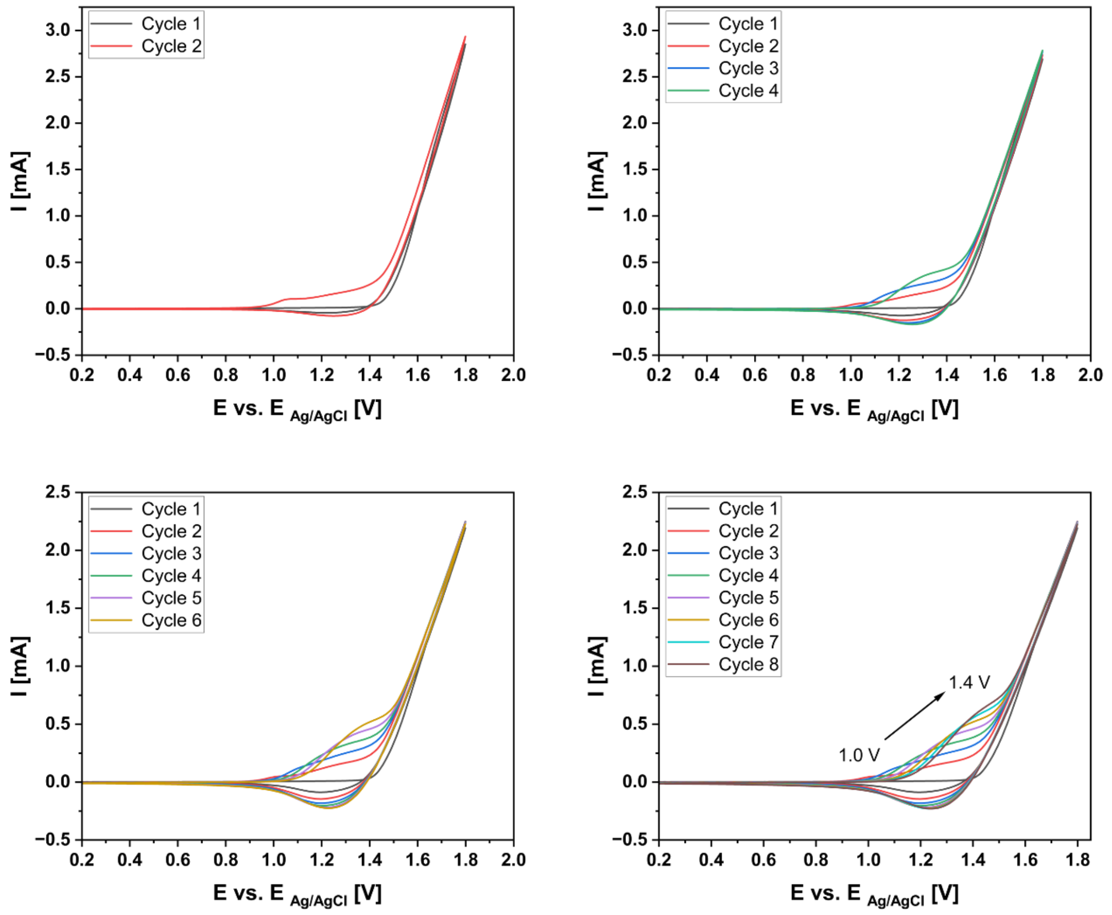

Figure S3: CV at FTO electrode, carried out for 1 mM solution of 4CzIPN in ACN:DCM (1:4) using 0.1 M NBu_4ClO_4 as electrolyte. Film synthesis done for 2 CV, 4 CV, 6 CV and 8 CV cycles.

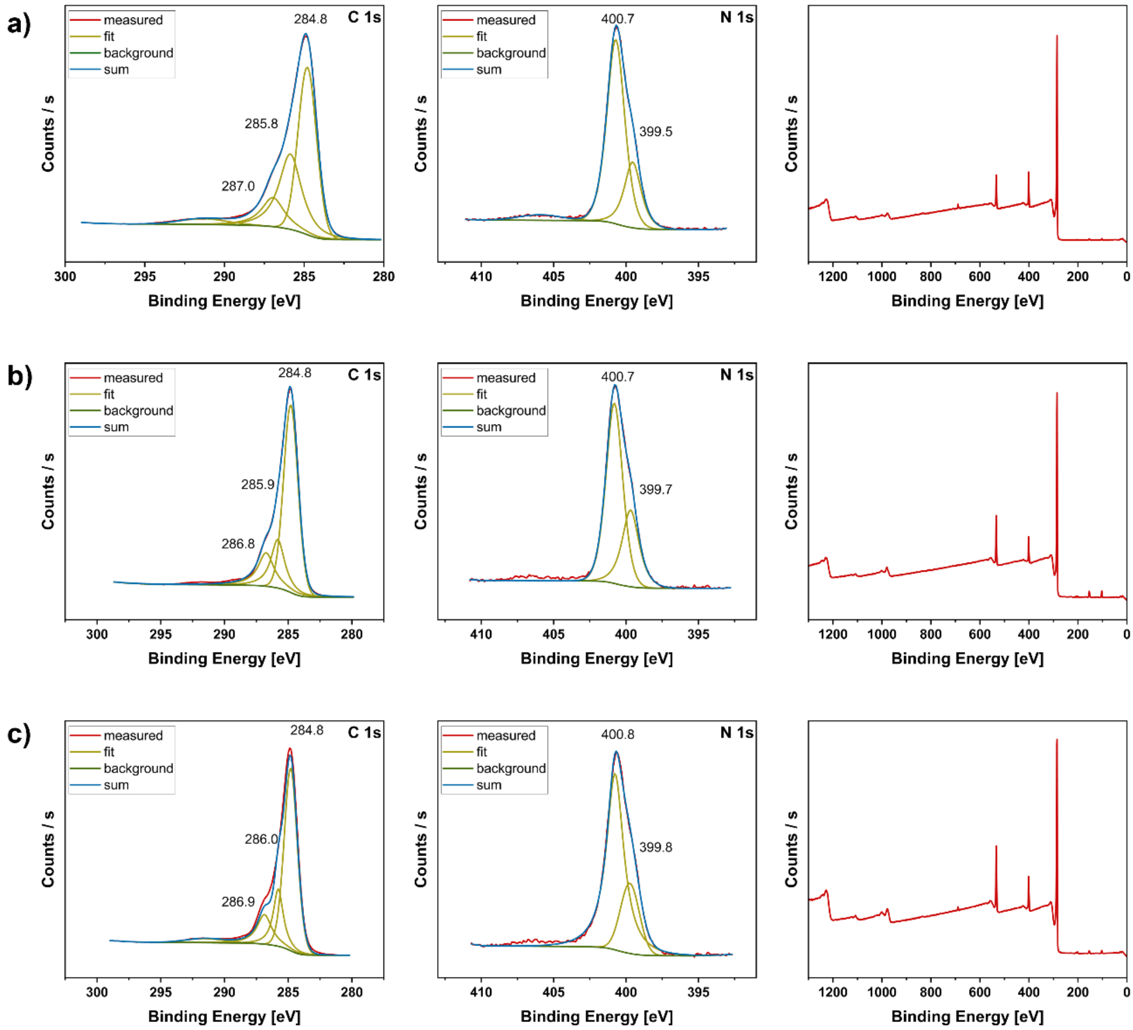

Figure S4: C 1s, N 1s and Survey XPS spectra of synthesized films after 2 (a), 4 (b) and 8 (c) CV cycles.

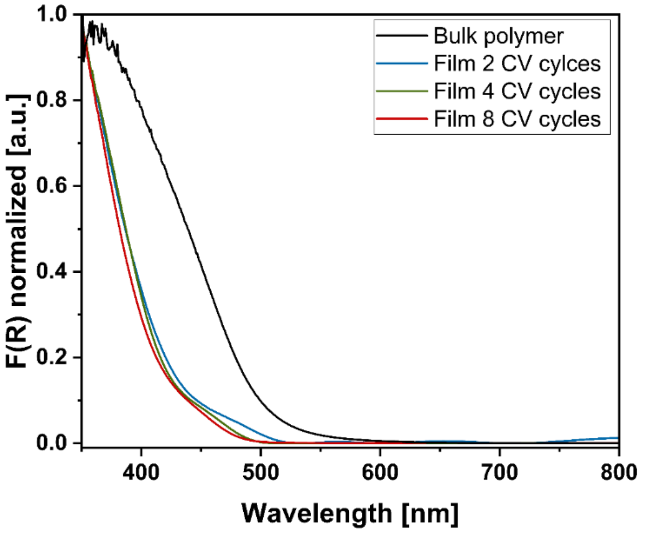

Figure S5: DR-UV-VIS spectrum of bulk polymer in comparison to films obtained after 2 (blue), 4 (green) and 8 (red) CV cycles.

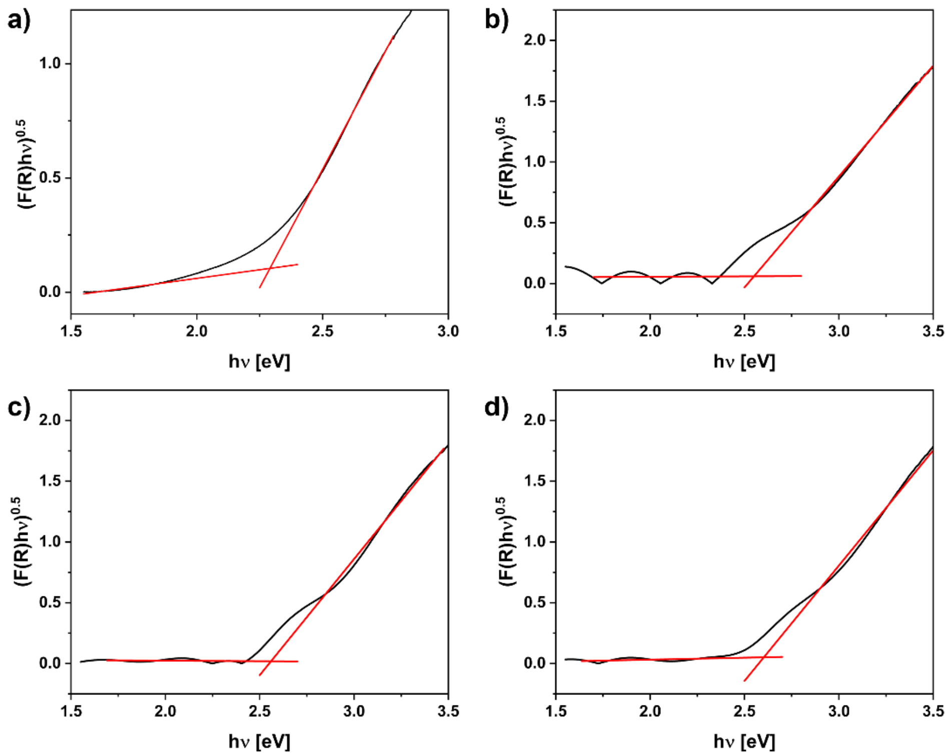

Figure S6: Tauc Plot of Bulk polymer (a) and film polymer after 2 (b), 4 (c) and 8 CV cycles (d).

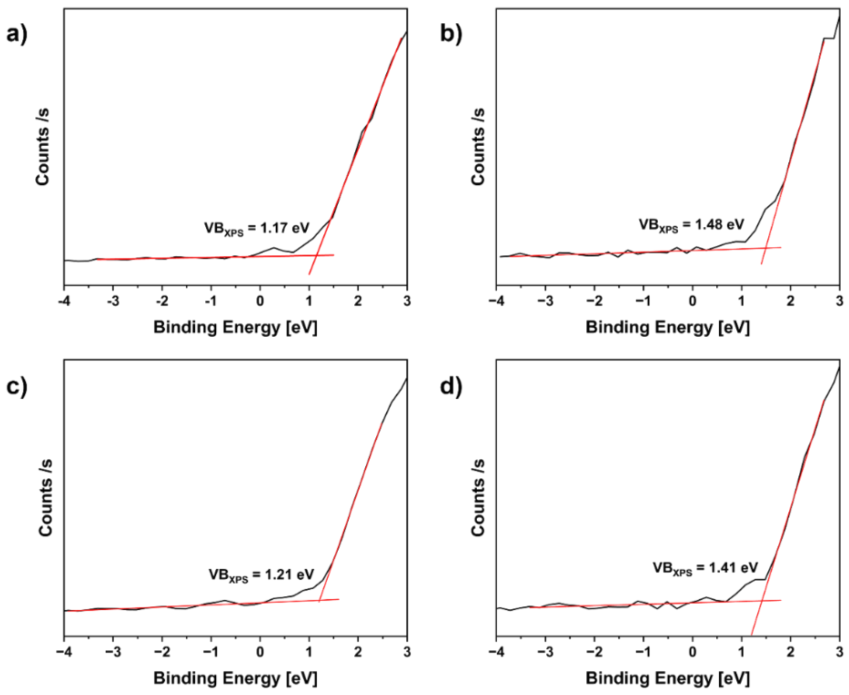

Figure S7: Valence Band XPS spectra of bulk polymer (a) and film polymer after 2 (b), 4 (c) and 8 CV cycles (d).

By fitting the linear parts of the onset of each VB XPS spectrum (Figure S7), the VB positions vs vacuum level were determined by using equation (1), where ϕ is the work function of the analyzer (4.35 eV), and summarized in table S1.

$$VB_{XPS} = -(\phi + XPS_{VB}) \qquad (1)$$

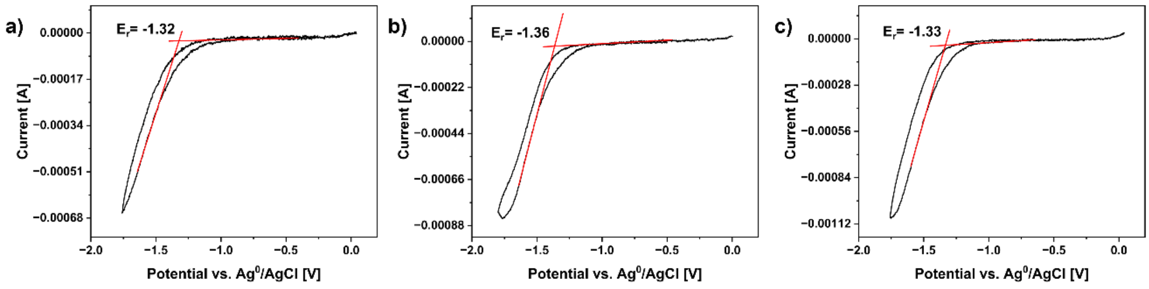

Figure S8: CVs (rate: 10 mV s-1 from 0 V to -1.8 V vs Ag/AgCl) of the films after 2 (a), 4 (b) and 8 (c) CV cycles carried out in 0.1 M $TABF_6$ in DCM.

Table S1: Comparison of CB level determined via electrochemistry and VB XPS of films in different thickness.

Film	$CB_{electro}$[a] vs. Ag^0/AgCl [V]	$CB_{electro}$[b] vs. NHE [V]	VB_{XPS} vs. Vac[c] [eV]	Bandgap[d] [eV]	CB_{XPS} vs. Vac[e] [eV]	CB_{XPS} vs. NHE[f] [V]	CB_{XPS} vs. $CB_{electro}$[g] [V]
2 CV	-1.32	-1.12	-5.81	2.56	-3.25	-1.19	-0.07
4 CV	-1.36	-1.16	-5.59	2.55	-3.04	-1.40	-0.24
8 CV	-1.33	-1.13	-5.76	2.60	-3.16	-1.28	-0.15

[a] *$CB_{electro}$ determined via CV (Figure S8).*

[b] *$CB_{electro}$ converted to NHE reference electrode.*

[c] *VB_{XPS} determined via VB XPS measurement (Figure S7) and equation (1).*

[d] *Bandgap determined via Tauc-Plot (Figure S6).*

[e] *Calculated CB_{XPS} level vs vacuum level by using VB_{XPS} and the bandgap: $CB_{XPS} = VB_{XPS}$ + bandgap*

[f] *CB_{XPS} level converted to NHE reference electrode.*

[g] *Comparison of electrochemical [b] and XPS determined [f] CB levels.*

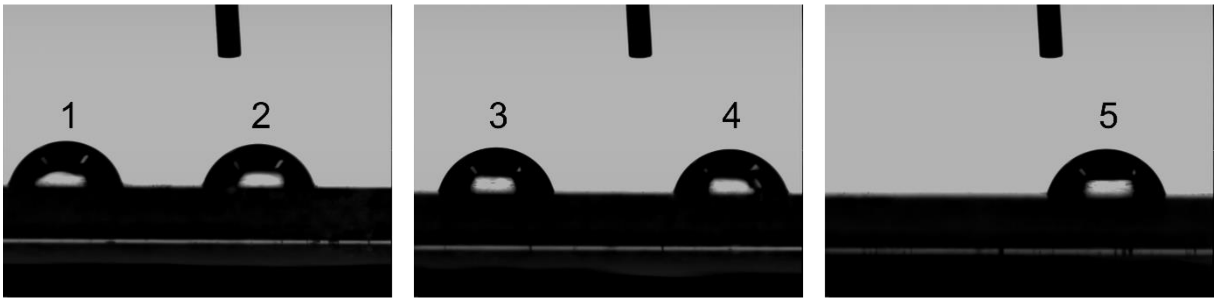

Figure S9: Contact angle measurement of milli-Q water (3 µL) onto film surface after 2 CV cycles.

Table S2: Determined contact angle between the base line and the tangent at the conic section curve at the three-phase contact point.

Water-droplet	Contact angle θ
1	78.8
2	81.9
3	77.1
4	75.2
5	75.5
**mean**	**77.7° ± 2.8°**

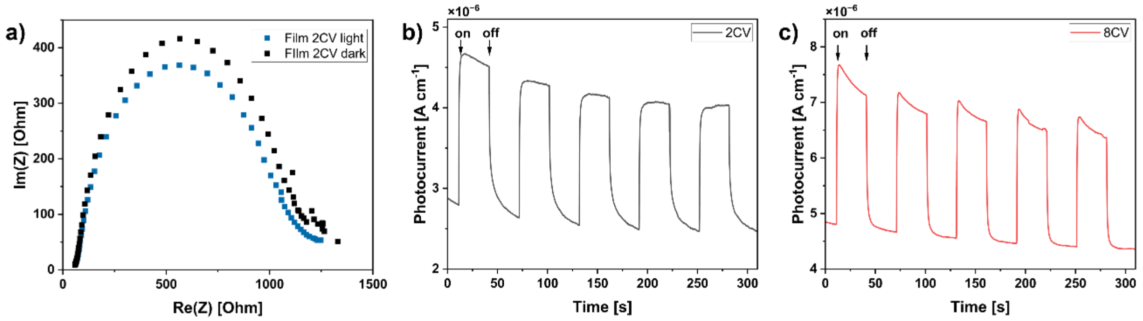

Figure S10: EIS of film after 2 CV cycles with and without illumination (a). Photocurrent of film after 2 (b) and 8 CV cycles (c). All measurements were conducted in 0.2 M Na_2SO_4 in ACN.

IV. Photocatalysis

All photocatalytic reactions were conducted in a two-neck quartz reactor. In case of bulk polymer, 5 mg of the powder was physically mixed by grinding. Afterwards it was dispersed in 16 mL of either 0.1 M ascorbic acid solution in water or in triethanolamine (TEOA) water mixture (1:4) and sonicated for 20 minutes. After the addition of 3 µL H_2PtCl_6 solution (8 wt%) the mixture was sonicated for additional 5 minutes and the reactor was sealed with rubber septa. Before irradiation the solution was degassed with argon for 20 minutes. Afterwards the reactor was placed in 15 cm distance to the 300 W Xe lamp (L.O.T. – Quantum Design) equipped with 400 nm long pass filter and irradiated at 20 °C under continuously stirring. After initial 15 minutes of Pt co-catalyst photo deposition, 1 mL of argon was injected and 1 mL of the gas phase was taken using a gas tight syringe every hour. Hydrogen was quantified by gas chromatography (FULI Instruments, GC9790II(PLF-01)) equipped with a thermal conductivity detector. The intensity of the light in the distance of 15 cm was measured using a Photometer (InternationalLight Model IL1400A).

In case of the polymer films 16 mL of TEOA water mixture (1:4) and 1 µL of H_2PtCl_6 solution (8 wt%) was mixed and placed in the photoreactor. Afterwards the regarding electrode coated with polymer film was placed in the photoreactor (film facing towards the light source). After degassing for 20 minutes with argon the film was illuminated without stirring the solution. Therefore, the time for photo deposition of the Pt co-catalyst was prolonged to 30 minutes. After that, every hour the produced hydrogen amount was quantified as described above.

The apparent quantum efficiency (AQE) was measured under the same conditions (TEOA as SA, Pt as co-catalyst, 20°C) as described above. The film or powder polymer was irradiated with a 365 nm LED from Thorlabs (M365LP1) in 5 cm distance. The intensity was measured ten times to be in average 126.22 W m^{-2}. The produced amount of hydrogen after a certain time was measured and the AQE was calculated by the following equation:

$$\mathrm{AQE} = \frac{2N_{\mathrm{H_2}}}{N_{\mathrm{Photon}}} \times 100\%$$

The amount of hydrogen molecules produced per second was calculated with the following equation, where n_{H2} is the amount of evolved gas within the irradiation time t and N_A is the Avogadro constant:

$$N_{H2} = \frac{n_{H2}}{t} \times N_A$$

The amount of incident photons N_{Photon} was calculated via the equation below, where P is the measured illumination power per area, A the irradiated area, λ the wavelength of the LED, h the Planck constant and c the speed of light.

$$N_{Photon} = \frac{P \times A \times \lambda}{h \times c}$$

In case of the polymer powder 0.1988 µmol was produced within 1 h in the illuminated area of 10.8 cm^2 and in case of the film 0.0133 µmol was produced within 2 h in the illuminated film area of 3 cm^2, yielding in AQEs of 2.7% and 0.3%, respectively.

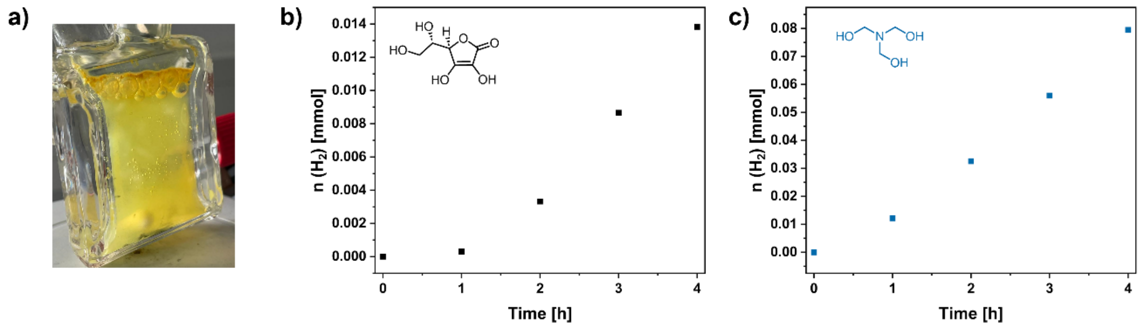

Figure S11: Image of bulk polymer after illumination (a). Time course of hydrogen production of bulk polymer using ascorbic acid (b) and triethanolamine (c) as sacrificial agent.

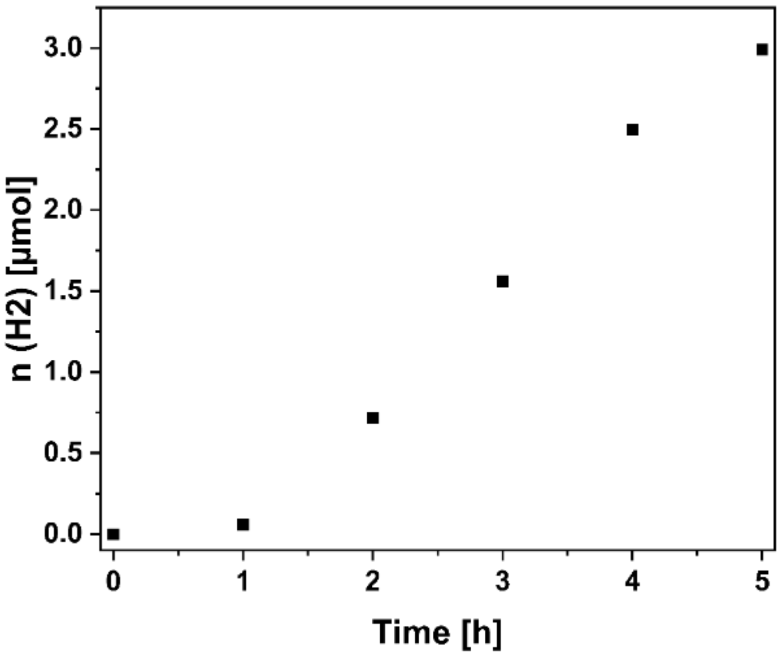

Figure S12: Time course of hydrogen production of bulk polymer without Pt co-catalyst under visible light (> 400 nm) in water/triethanolamine mixture (4:1).

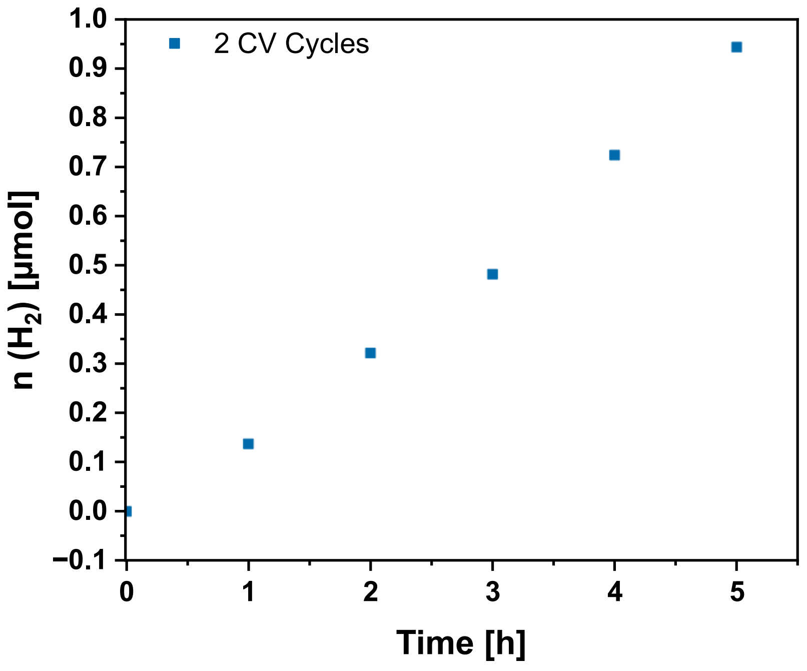

Figure S13: Time course of photocatalytic hydrogen production of 4CzIPN polymer films after 2 CV cycles (film area: 3 cm^2) under visible light (> 400 nm) in water/triethanolamine mixture (4:1) and Pt as co-catalyst.

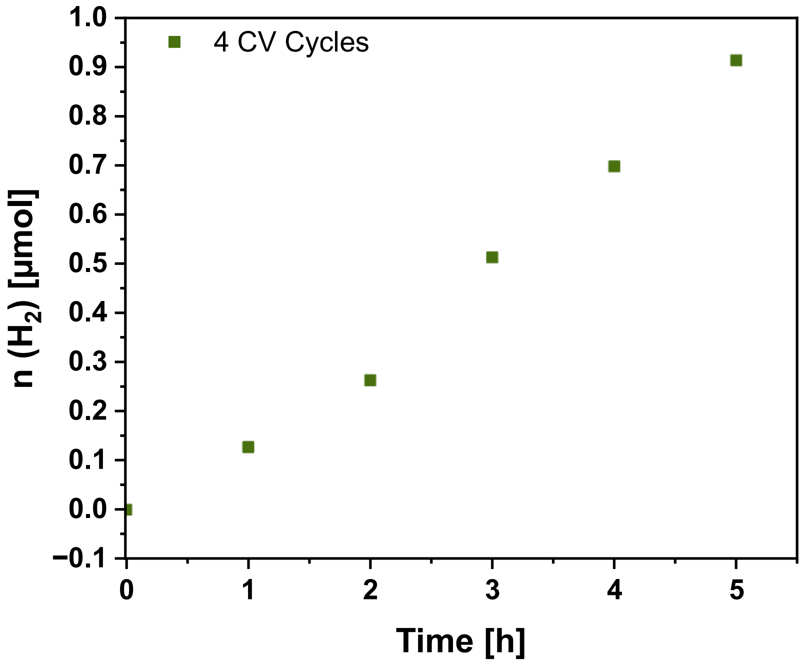

Figure S14: Time course of photocatalytic hydrogen production of 4CzIPN polymer films after 4 CV cycles (film area: 3.2 cm^2) under visible light (> 400 nm) in water/triethanolamine mixture (4:1) and Pt as co-catalyst.

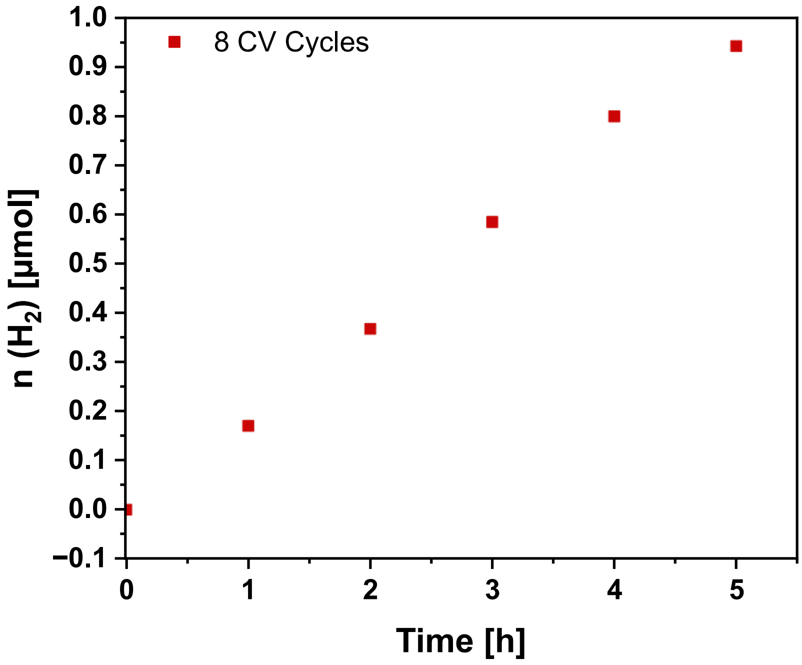

Figure S15: Time course of photocatalytic hydrogen production of 4CzIPN polymer films after 8 CV cycles (film area: 3 cm^2) under visible light (> 400 nm) in water/triethanolamine mixture (4:1) and Pt as co-catalyst.

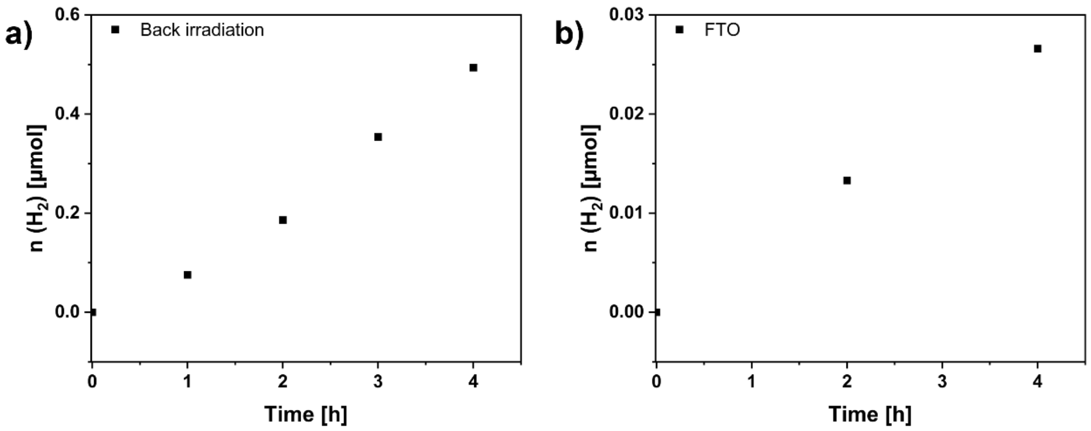

Figure S16: Time course of hydrogen production of 8 CV film (film area: 3 cm^2) back irradiated (a) and blank FTO electrode under visible light (> 400 nm) in water/triethanolamine mixture (4:1) and Pt as co-catalyst.

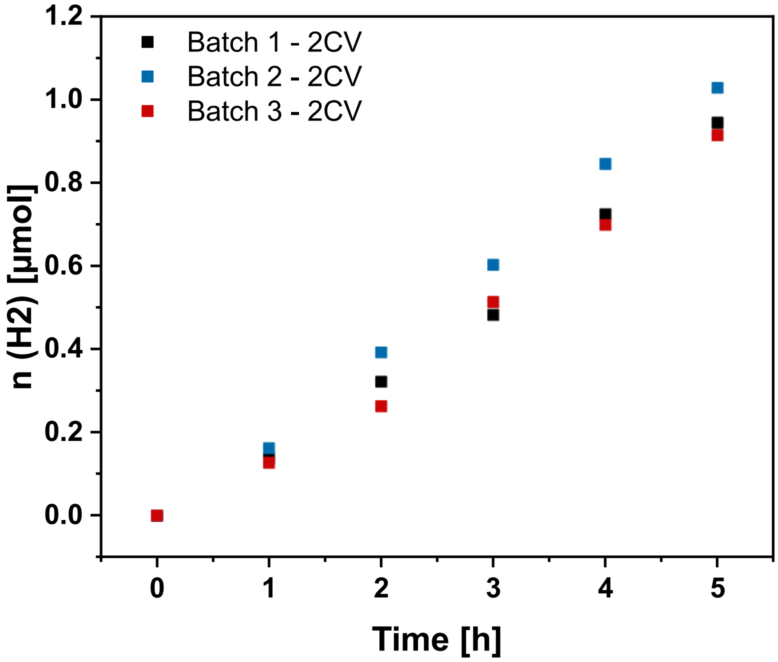

Figure S 17: Comparison of time course of hydrogen production of different batches of 2 CV film (film area of each: 3 cm^2) under visible light (> 400 nm) in water/triethanolamine mixture (4:1) and Pt as co-catalyst.

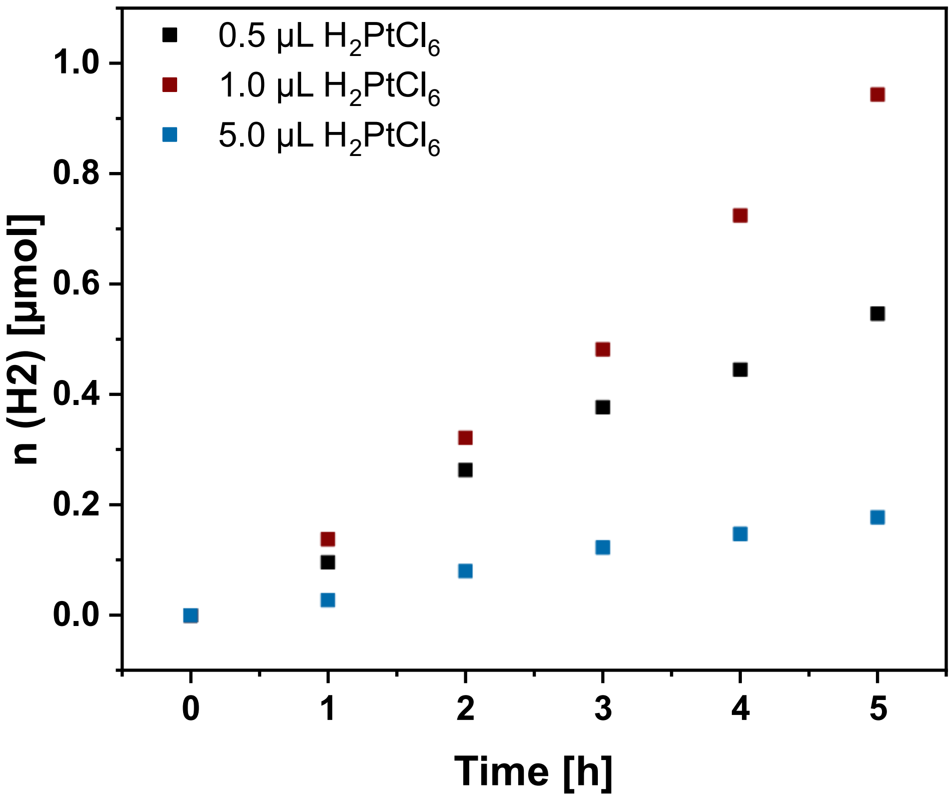

Figure S18: Comparison of time course of hydrogen production of 2CV films (film area of each: 3 cm^2) with different amounts of Pt co-catalyst under visible light irradiation (> 400 nm) in water/triethanolamine mixture (4:1).

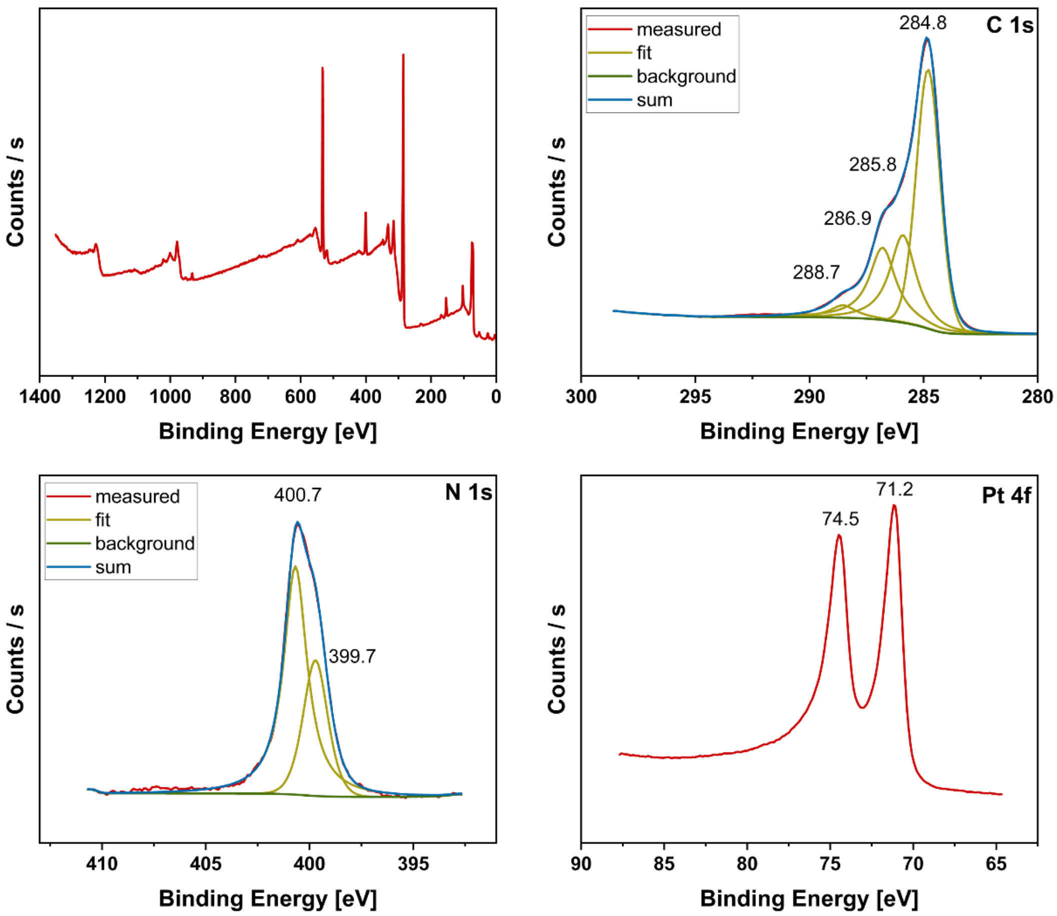

Figure S19: XPS analysis of 2 CV cycles film after photocatalytic recycling test.

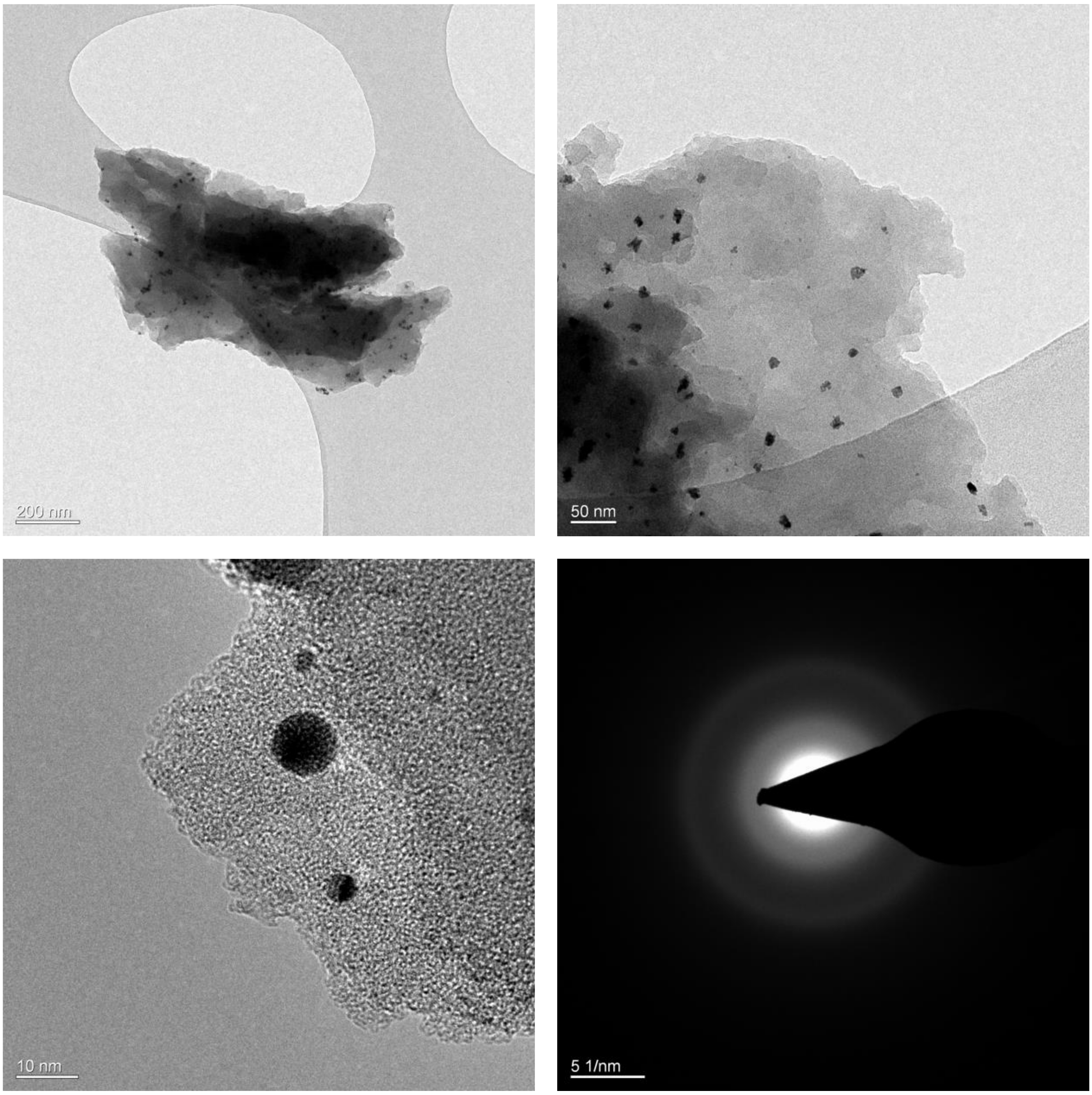

Figure S20: Transmission electron microscopy analysis of 2CV film scratched off the electrode after catalysis and diffraction pattern captured on the particle top left.

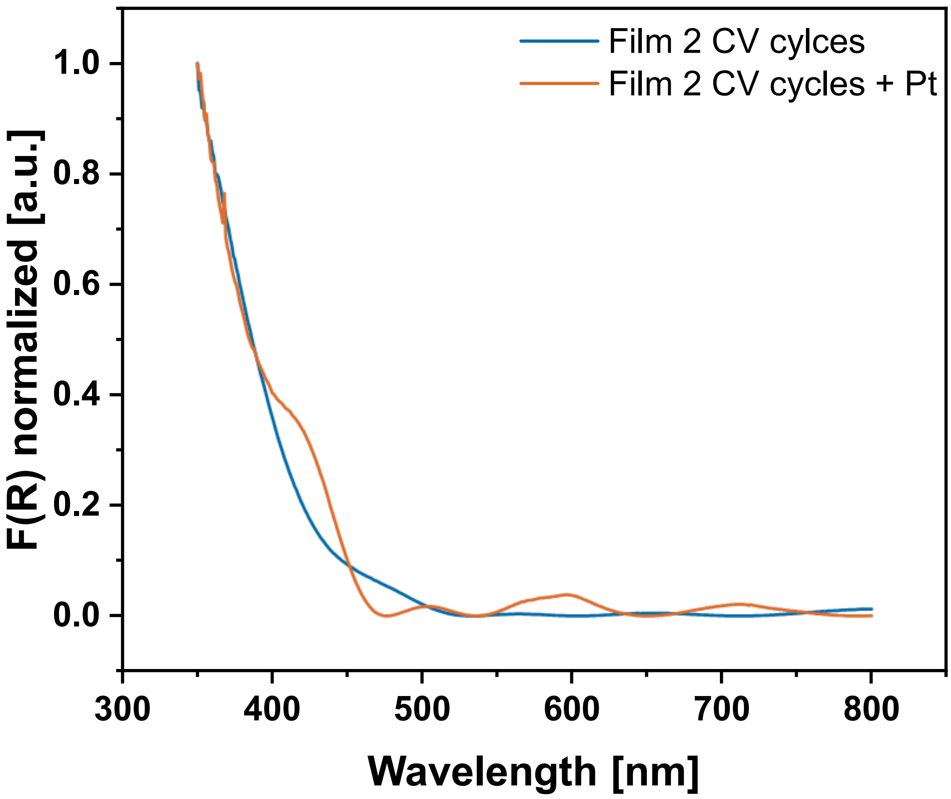

Figure S21: DR-UV-VIS spectrum of polymer film after 2 CV cycles before (blue) and after photo-deposition of Pt co-catalyst (orange).

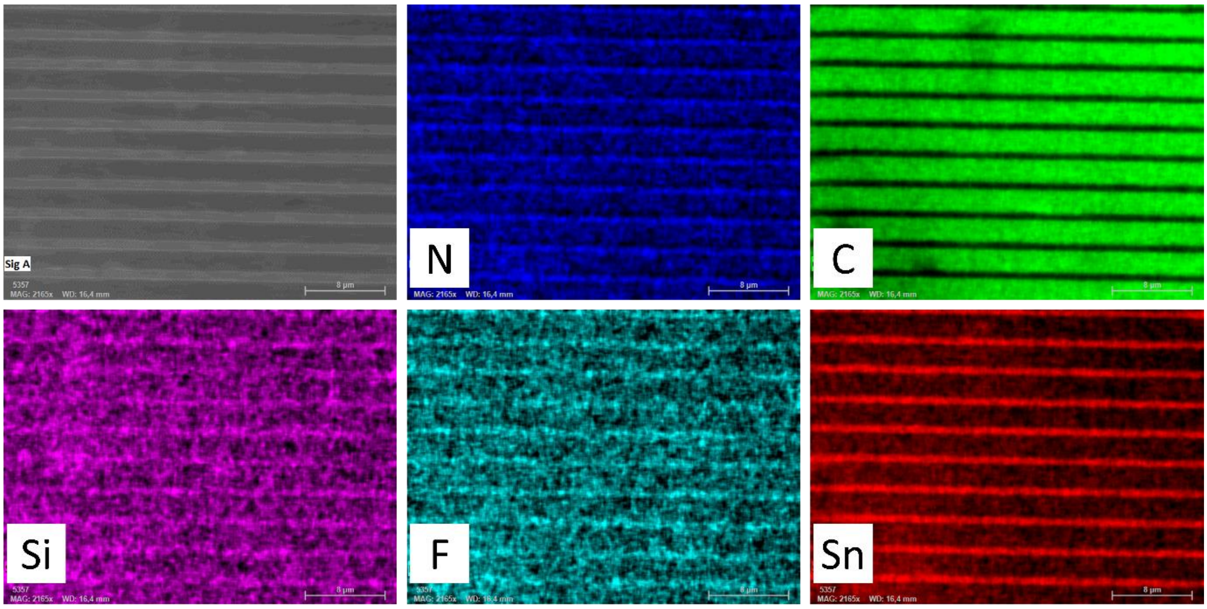

Figure S22: SEM EDX analysis of post synthetic microstructured polymer film.

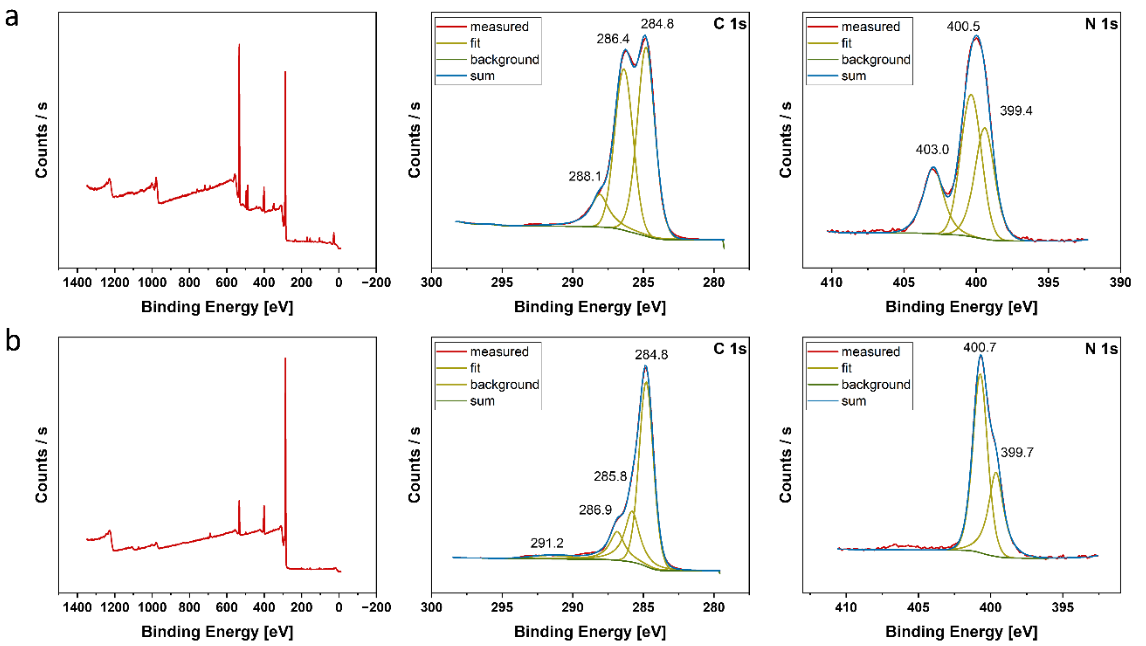

Figure S23: XPS analysis of post synthetic microstructured film (a) and film synthesized on microstructured FTO electrode (b).

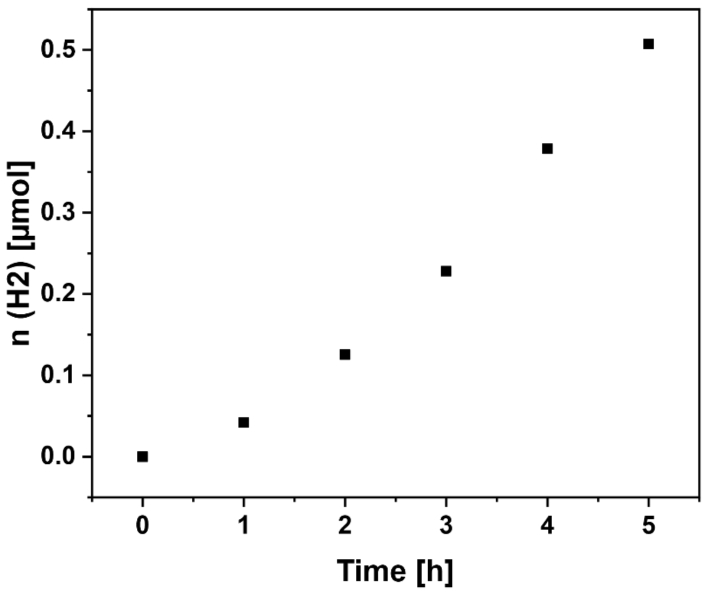

Figure S24: Time course of hydrogen production of a post-synthetic microstructured polymer film (film area 2.8 cm^2).

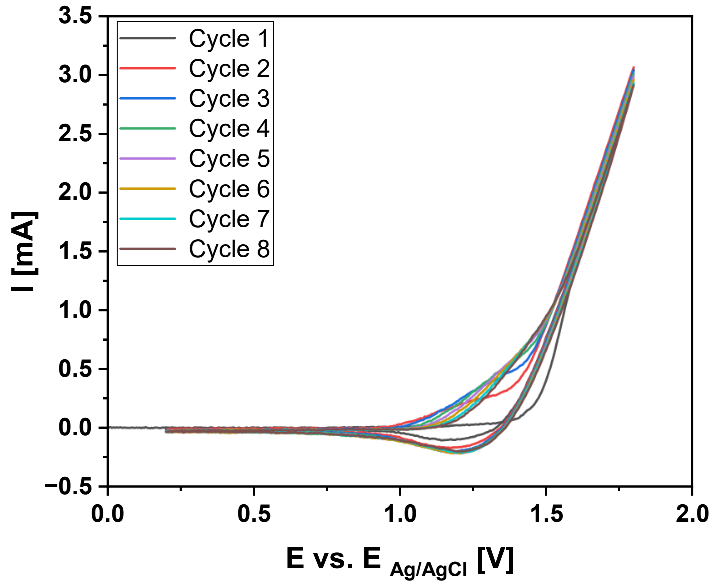

Figure S25: Film synthesis via CV at sFTO electrode, carried out for 1 mM solution of 4CzIPN in ACN:DCM (1:4) using 0.1 M NBu_4ClO_4 as electrolyte.

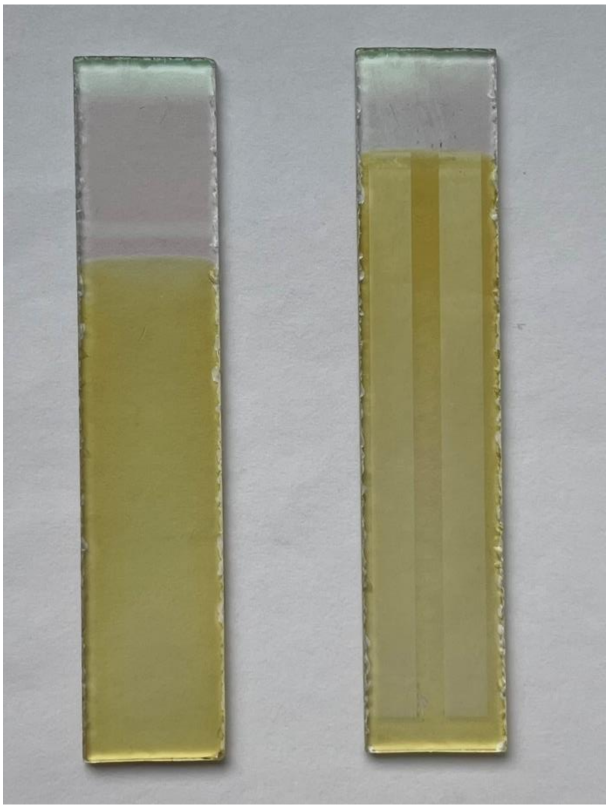

Figure S26: Photograph of films after 8 CV cycles on FTO electrode (left) and laser-ablated microstructured FTO electrode (right).

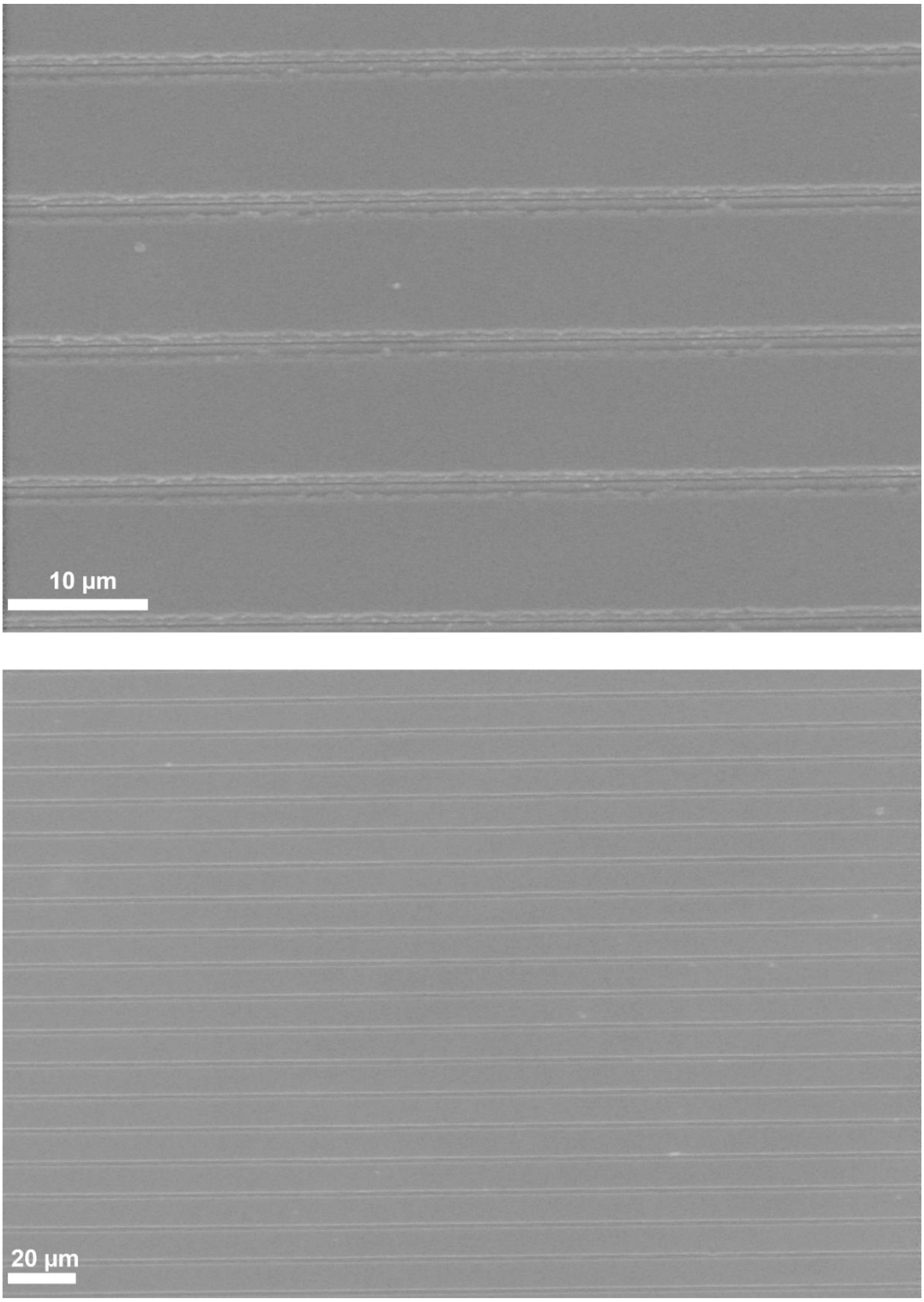

Figure S27: SEM EDX analysis of the film synthesized on a microstructured FTO electrode.
